# Ontogeny of Carbon Monoxide-Related Gene Expression in a Deep-Diving Marine Mammal

**DOI:** 10.3389/fphys.2021.762102

**Published:** 2021-10-21

**Authors:** Elizabeth R. Piotrowski, Michael S. Tift, Daniel E. Crocker, Anna B. Pearson, José P. Vázquez-Medina, Anna D. Keith, Jane I. Khudyakov

**Affiliations:** ^1^Department of Biological Sciences, University of the Pacific, Stockton, CA, United States; ^2^Department of Biology and Marine Biology, University of North Carolina Wilmington, Wilmington, NC, United States; ^3^Biology Department, Sonoma State University, Rohnert Park, CA, United States; ^4^Department of Integrative Biology, University of California, Berkeley, Berkeley, CA, United States

**Keywords:** hypoxia tolerance, marine mammal, gene expression, carbon monoxide, heme oxygenase, diving physiology

## Abstract

Marine mammals such as northern elephant seals (NES) routinely experience hypoxemia and ischemia-reperfusion events to many tissues during deep dives with no apparent adverse effects. Adaptations to diving include increased antioxidants and elevated oxygen storage capacity associated with high hemoprotein content in blood and muscle. The natural turnover of heme by heme oxygenase enzymes (encoded by *HMOX1* and *HMOX2*) produces endogenous carbon monoxide (CO), which is present at high levels in NES blood and has been shown to have cytoprotective effects in laboratory systems exposed to hypoxia. To understand how pathways associated with endogenous CO production and signaling change across ontogeny in diving mammals, we measured muscle CO and baseline expression of 17 CO-related genes in skeletal muscle and whole blood of three age classes of NES. Muscle CO levels approached those of animals exposed to high exogenous CO, increased with age, and were significantly correlated with gene expression levels. Muscle expression of genes associated with CO production and antioxidant defenses (*HMOX1*, *BVR*, *GPX3*, *PRDX1*) increased with age and was highest in adult females, while that of genes associated with protection from lipid peroxidation (*GPX4*, *PRDX6*, *PRDX1*, *SIRT1*) was highest in adult males. In contrast, muscle expression of mitochondrial biogenesis regulators (*PGC1A*, *ESRRA*, *ESRRG*) was highest in pups, while genes associated with inflammation (*HMOX2*, *NRF2*, *IL1B*) did not vary with age or sex. Blood expression of genes involved in regulation of inflammation (*IL1B*, *NRF2*, *BVR*, *IL10*) was highest in pups, while *HMOX1*, *HMOX2* and pro-inflammatory markers (*TLR4*, *CCL4*, *PRDX1*, *TNFA*) did not vary with age. We propose that ontogenetic upregulation of baseline *HMOX1* expression in skeletal muscle of NES may, in part, underlie increases in CO levels and expression of genes encoding antioxidant enzymes. *HMOX2*, in turn, may play a role in regulating inflammation related to ischemia and reperfusion in muscle and circulating immune cells. Our data suggest putative ontogenetic mechanisms that may enable phocid pups to transition to a deep-diving lifestyle, including high baseline expression of genes associated with mitochondrial biogenesis and immune system activation during postnatal development and increased expression of genes associated with protection from lipid peroxidation in adulthood.

## Introduction

While intermittent hypoxemia, ischemia, and reperfusion cause tissue damage in laboratory animals and humans, marine mammals routinely experience these conditions due to their diving lifestyle with no apparent adverse effects (Allen and Vázquez-Medina, 2019). Physiological adaptations of marine mammals to breath-hold diving include enhanced blood oxygen stores and high antioxidant capacity (Ponganis, 2011). For example, deep-diving phocid seals such as the northern elephant seals (*Mirounga angustirostris;* NES) have some of the highest reported mass-specific blood volumes and total body oxygen stores in mammals (Kooyman and Ponganis, 1998). Elevated total oxygen stores and large body sizes contribute to greater breath-hold capacity and longer dive durations (up to 90 min) in NES compared to many other pinniped species (Hassrick et al., 2010). NES spend more than 90% of their time submerged at sea, undergoing repeated apneas, peripheral vasoconstriction, and bradycardia as part of the dive response (Andrews et al., 1997; Ponganis et al., 2008). *In vivo* blood oxygen measurements in diving NES have revealed over 90% depletion of blood oxygen stores during routine dives (Meir et al., 2009), further highlighting the remarkable hypoxemia tolerance in this elite-diving species. Even when hauled out on land, NES undergo repeated sleep apneas with little to no apparent oxidative damage to tissues (Stockard et al., 2007; Vázquez-Medina et al., 2011a; Tift et al., 2013).

Despite new advances in understanding cellular and molecular mechanisms underlying hypoxia tolerance in deep-diving species, few studies have addressed their mechanistic drivers within an ontogeny framework (Weitzner et al., 2020). Among marine mammals, pinnipeds are unique in their developmental transition from birth and nursing on land to diving after weaning. This makes them ideal model systems for studying ontogeny of diving adaptation. In general, blood and muscle oxygen stores (hemoglobin and myoglobin, respectively) increase as marine mammals approach adulthood, enhancing their dive capacity (Horning and Trillmich, 1997; Dolar et al., 1999). In Steller sea lions (*Eumetopias jubatus*), mass-specific total body oxygen stores not only increase with age, but shift predominantly to muscle for primary oxygen storage (Richmond et al., 2006). Similar age-related changes in oxygen-storing capacity have been observed in other pinniped species, such as the Weddell seal (*Leptonychotes weddellii*) (Penso-Dolfin et al., 2020). Expression and activity of antioxidant enzymes were also found to increase with maturation in hooded seals (*Cystophora cristata*) and ringed seals (*Pusa hispida*) (Elsner et al., 1998; Vázquez-Medina et al., 2006, 2011b). Under basal conditions, antioxidant enzyme activity was more than fourfold higher in skeletal muscle of adult hooded seals compared to newborn and weaned pups (Vázquez-Medina et al., 2011c). High antioxidant expression and activity have also been reported in northern NES, specifically in the context of adaptive responses to apneas and prolonged fasting (Allen and Vázquez-Medina, 2019; Ensminger et al., 2021), but have not been extensively profiled across ontogeny.

One potential driver of cytoprotective gene expression in mammals exposed to chronic hypoxia is carbon monoxide (CO), a gasotransmitter produced during heme catabolism by heme oxygenase (HO) enzymes. NES and Weddell seals have levels of blood CO that are comparable to chronic human smokers and that increase with age and blood oxygen stores (Pugh, 1959; Tift et al., 2014). Humans living at high altitude in South America also exhibit elevated hemoglobin concentrations in blood, and high blood CO (Tift et al., 2020). Recent studies in humans and laboratory rodents have shown that exogenous administration of low to moderate doses of CO via inhalation or pharmacological application of CO-releasing molecules (CO-RMs) stimulates mitochondrial biogenesis, regulates inflammation, and confers cytoprotection from injuries associated with hypoxia and ischemia-reperfusion and cold ischemia during organ transplantation (Motterlini and Otterbein, 2010; Dugbartey, 2021). The cellular effects of CO are mediated in part by the p38-MAPK, soluble guanylyl cyclase, and NFkB signaling pathways and their downstream targets (Ryter, 2020). CO administration in mice increases expression of *PGC1A* and *SIRT1*, which regulate mitochondrial biogenesis, oxidative metabolism, and cell survival (Suliman et al., 2007; Rhodes et al., 2009; Kim et al., 2015; Sun et al., 2017). CO treatment under inflammatory conditions also decreases levels of pro-inflammatory cytokines TNF-α, IL-1β, and MIP-1β (*CCL4*) and inhibits TLR4 and JNK signaling, while increasing production of the anti-inflammatory cytokine IL-10 (Otterbein et al., 2000; Ryter et al., 2018). Currently, NES and Weddell seals are the only non-laboratory species confirmed to endogenously produce CO at concentrations believed to have cytoprotective effects (Pugh, 1959; Tift et al., 2014). However, the regulation of the HO/CO pathway and downstream effects of elevated CO in diving mammals have not been examined.

Carbon monoxide is produced endogenously by the heme degradation activity of two HO isoforms, HO-1 and HO-2 (encoded by the genes *HMOX1* and *HMOX2*, respectively). While *HMOX2* is considered constitutively expressed, *HMOX1* expression is induced by a number of stimuli, including hypoxia, oxidative stress and inflammation, and is regulated by the electrophile-responsive transcription factor Nrf2 (Gozzelino et al., 2010). In addition to CO, heme catabolism by HO enzymes generates biliverdin, which is converted by biliverdin reductase (BVR) to bilirubin, a potent antioxidant at low to moderate concentrations (Jansen and Daiber, 2012; Chen et al., 2018). Moreover, HO-1 may exert direct cytoprotective effects by translocating to the nucleus and activating expression of transcription factors that regulate cell proliferation and survival (Lin et al., 2007). HO-2 has also been implicated in cellular responses to hypoxia and oxygen sensing in the carotid body in humans and rodents (Muñoz-Sánchez and Chánez-Cárdenas, 2014), and has shown evidence of positive selection in human populations adapted to high altitude (Simonson et al., 2010). However, the expression and potential role of HO enzymes in hypoxia tolerance in wild animals have not yet been studied.

Due to their high levels of endogenous CO in blood, NES are a natural study system for examining the role of CO in hypoxia tolerance. In this study, we evaluated ontogenetic changes in gene expression associated with CO production and signaling in NES skeletal muscle and whole blood, two tissues which are routinely sampled from wild NES. Skeletal muscle experiences myoglobin desaturation and some ischemia and reperfusion during apneas in NES (Ponganis et al., 2002, 2008). It also contains high abundance of hemoproteins (myoglobin and cytochromes), which could serve as potential sources of CO production. Gene expression in whole blood, which also experiences fluctuations in oxygen levels (Meir et al., 2009), reflects immune activity as it is derived primarily from peripheral blood mononuclear cells such as lymphocytes and monocytes (He et al., 2019). We measured CO levels and expression of 13 genes in skeletal muscle and 10 genes in whole blood of weaned pups, juveniles, and adult NES. Genes targeted in muscle encoded proteins associated with CO production (*HMOX1*, *HMOX2*, *BVR*), antioxidant defense (*NRF2*, *GPX3*, *GPX4*, *PRDX1, PRDX6*, *SIRT1*), mitochondrial biogenesis (*PGC1A*, *ESRRA*, *ESRRG*), and inflammation (*IL1B*). Genes targeted in whole blood included six markers measured in muscle (*HMOX1*, *HMOX2*, *BVR*, *NRF2*, *PRDX1*, *IL1B*) and four others associated with inflammation (*IL10, TNFA*, *TLR4*, *CCL4*). We examined whether baseline gene expression varied with age class, sex, and fasting state, as NES were sampled early and late in haul-out periods characterized by extensive fasting, which can impact gene activity (Champagne et al., 2012). We hypothesized that expression of genes associated with CO production, signaling, and cytoprotective effects is correlated with CO levels and increases with age and dive capacity in NES, providing a potential mechanism of ontogeny of ischemia-reperfusion tolerance in a deep-diving mammal.

## Materials and Methods

### Study Subjects

All animal handling procedures were approved by Sonoma State University and University of the Pacific Institutional Animal Care and Use Committees and conducted under National Marine Fisheries Service permit nos. 19108 and 23188. Three age classes of NES were sampled at Año Nuevo State Reserve (San Mateo County, CA, United States): weaned pups (*n* = 15; 5 females and 10 males, spring 2020), juveniles (*n* = 9; 4 females, 4 males, 1 sex not recorded, fall 2017), and molting adults (*n* = 22; 11 females and 11 males, summer 2020 and 2021; Supplementary Table 1). NES pups fast for 6–8 weeks at the rookery after weaning before leaving for their first foraging trip. Adults haul out for approximately 4 weeks to molt, during which they are also fasting (Champagne et al., 2012). In contrast, juveniles (0.8–1.8 year-old) haul out for a brief period in the fall that is not characterized by extensive fasting (Jelincic et al., 2017). To examine whether any of the markers measured in this study varied with fasting, independent cohorts of weaned pups and adults were sampled at the beginning and end of their haulout periods. Seven pups were sampled early in their post-weaning fast, while 8 were sampled after 4–6 weeks of fasting. Six adult females and 5 adult males were sampled during early molting (<10% molted), and 5 females and 6 males were sampled during late molting (>90% molted, after 3–4 weeks of fasting). Blood samples were not obtained for 2 of the weaned pups, 2 of the adult females, or any of the juveniles. CO concentrations in skeletal muscle were not obtained for 2 of the weaned pups, one of the juveniles, one of the adult females, and one of the adult males.

### Sample Collection

Collection of skeletal muscle and whole blood samples was conducted after study animals were anesthetized as described previously (Tift et al., 2014). Specifically, animals were chemically immobilized with 1 mg/kg intramuscular injections of tiletamine-zolazepam HCl (Telazol, Fort Dodge Animal Health, United States), and sedation was sustained with intravenous doses of ketamine and diazepam (0.25–1 mg/kg) (Fort Dodge Animal Health, IA, United States). Blood samples were obtained from the extradural vein using an 18 G, 3.25-inch spinal needle. Whole blood (2.5 ml) was drawn into PAXgene^TM^ Blood RNA Tubes (PreAnalytiX, United States), mixed, stored for ∼ 2 h at room temperature, frozen at −20°C for 24 h, and transferred to −80°C for long-term storage. Skeletal muscle samples were collected from the external abdominal oblique muscle using a 6.0-mm diameter biopsy punch (Integra Miltex, United States), frozen in a cryovial on dry ice and stored at −80°C until further processing. Muscle biopsies collected from juveniles were preserved in RNA*later*^TM^ Stabilizing Solution (∼300 mg tissue per 1.5 mL; Invitrogen, United States) for 24 h at 4°C. RNA*later*^TM^ was removed from samples prior to freezing at −80°C for long term storage. We previously showed that the integrity of RNA isolated from samples frozen in the field and those preserved in RNA*later*^TM^ were comparable and can be used interchangeably for targeted gene expression analyses (Pujade Busqueta et al., 2020).

### Carbon Monoxide Measurements

CO extraction and quantification from tissues were conducted using previously established protocols (Vreman et al., 2005). The quantity of CO extracted from tissue samples was measured using a reducing compound photometer gas chromatography system (GC RCP, Peak Performer 1, Peak Laboratories LLC, United States). A certified calibration gas (1.02 ppm CO balanced with nitrogen) was purchased from Airgas^®^ and used to generate a daily standard curve prior to experiments. Amber borosilicate glass chromatography vials (2 mL) with gas-tight silicone septa were used for all experiments and were purged of CO using a custom catalytic converter prior to the addition of calibration gas or samples. The vial headspace was flushed with CO-free carrier gas into the gas chromatography system via a custom-made double needle assembly attached to the front of the instrument. Frozen aliquots of tissue sample were rinsed of external blood with ice-cold KH_2_PO_4_ buffer (pH 7.4) and placed into 2.5 mL microcentrifuge tubes. Tissues were diluted to approximately 10–45% (w/w) with ice-cold Milli-Q^®^ water. The tissue was thoroughly diced with surgical scissors over ice and then homogenized using the Ultra-Turrax T8 grinder (IKA Works, Inc., United States) for 6 to 8 one-sec pulses followed by 6 to 8 one-sec pulses from an ultrasonic cell disruptor (Branson, Danbury, CT, United States). After completely homogenized, 10 μL of tissue homogenate and 20 μL of 20% sulfosalicylic acid were injected into the purged 2 mL amber vials with gas-tight syringes connected to repeating dispensers. The CO released into the headspace of the amber vials was measured using the system mentioned previously. Each sample was run in triplicate. CO values were reported as pmol^∗^mg^–1^ wet weight tissue.

### RNA Isolation

Muscle tissue (30–60 mg) was minced using a scalpel on dry ice and homogenized by bead beating in 1 mL of TRIzol Reagent (Life Technologies, United States) using a Bullet Blender Storm 24 instrument (Next Advance, United States) at max power for three two-min cycles. Tissue homogenates were further disrupted using a 1 mL syringe and 22 G needle to shear genomic DNA and centrifuged to pellet insoluble components. RNA was isolated from homogenates by phase extraction using chloroform (VWR Life Sciences, United States) following the TRIzol manufacturer’s protocol. RNA was purified using the RNeasy Mini kit (Qiagen, United States) according to the manufacturer’s protocol, including a 15-min on-column DNase I digest. Total RNA was isolated from whole blood using the PAXgene Blood RNA Kit Version 2 (PreAnalytiX, United States) following the manufacturer’s protocol with a 15-min on-column DNAse I digest. RNA concentration was quantified using Qubit 3.0 Fluorometer Broad Range RNA Assay (Life Technologies, United States). RNA integrity was assessed using the Total RNA 6000 Pico Kit on the 2100 Bioanalyzer (Agilent Technologies, United States). Mean (±S.D.) RNA integrity numbers (RIN) for muscle and blood samples were 8.93 ± 0.37 and 5.11 ± 1.26, respectively. RIN values for RNA isolated from muscle samples that were preserved in RNA*later*^TM^ and those that were flash frozen in liquid nitrogen were comparable as shown previously (Pujade Busqueta et al., 2020).

### RT-Quantitative Polymerase Chain Reaction

Total RNA (0.5 μg input) was reverse transcribed to complementary DNA (cDNA) using SuperScript IV VILO Master Mix with ezDNase (Thermo Fisher, United States). cDNA samples were diluted 1:10 and 2 μL were used in a 20 μL real-time quantitative polymerase chain reaction (qPCR) with PowerUp SYBR Green Master Mix (Thermo Fisher, United States). qPCR was performed on a QuantStudio 5 Real-Time PCR System instrument (Thermo Fisher, United States) using the following program: 2 min at 50°C, 2 min at 95°C and 40 cycles of 15 s at 95°C and 60 s at 60°C. All samples were run in triplicate with intra-assay coefficient of variation (CV) < 0.5% and inter-assay CV < 1%. No-template and minus-RT controls were included in each run and did not show any amplification.

Sequence-specific primers were designed with NCBI Primer-Blast using NES transcriptomes (Supplementary Table 2) (Khudyakov et al., 2015; Deyarmin et al., 2019). Eighteen target genes were selected based on significant BLASTX hits to protein orthologs in the UniProt SwissProt database (*e*-value < 0.001) and high transcript abundance in transcriptomes (transcripts per million, TPM ≥ 20). Primers were designed to specifically target highly conserved regions of each transcript. All primers were used at 400 nM with the exception of *EF2* and *GAPDH*, which were used at 200 nM final concentration. Amplification efficiencies for each primer pair (Supplementary Table 2) were calculated from slopes of standard curves of serially 1:2-diluted NES cDNA (*n* = 6 dilutions). Primer specificity was confirmed using melt curve analysis and agarose gel electrophoresis.

Normalized gene expression values (delta C_T_) were obtained by subtracting the C_T_ of the gene of interest from the C_T_ of a reference gene (Schmittgen and Livak, 2008). *EF2* was used as the reference gene for muscle, while *GAPDH* was used as the reference gene for blood after evaluation of stability using RefFinder (CV: muscle *EF2* = 2.45%, blood *GAPDH* = 2.58%) (Xie et al., 2012). Delta C_T_ values and primer efficiencies were calculated using Microsoft Excel 2020.

### Statistical Analyses

Data analyses were conducted using R v3.6.2 in RStudio v1.3.1073 (R Core Team, 2016, 2019). Spearman correlation (*r*_*S*_) was conducted using the ‘corr.test’ function from the *psych* package (Revelle, 2019) to evaluate associations between normalized expression levels of target genes. Gene expression data were scaled across samples and summarized using the *pheatmap* package (Kolde, 2019) with complete clustering by row and column (Euclidean distance).

Principal components analysis (PCA) of muscle and blood gene expression levels was conducted using the ‘principal’ function in the *psych* package (Revelle, 2019) after evaluating the appropriateness of this approach (Bartlett’s test of sphericity *p* < 0.05, determinant > 0.00001, Kaiser-Meyer-Olkin mean sampling adequacy values > 0.7) (Field et al., 2012). PCA diagnostic data are shown in Supplementary Table 3. The presence of potential outliers was assessed using Mahalanobis distance. No outliers in the muscle or blood gene expression datasets were identified at the recommended chi-square cutoff of alpha = 0.01 (Leys et al., 2018). Principal component interpretability was improved using varimax rotation.

Rotated components were extracted, and general linear models (GLM) were used to examine whether they varied by age, sex, fasting state (early, late) and their interactions. The ‘Anova’ function in the *car* package was used to obtain type III sums of squares. We first ran full models including all main and interaction terms, after which non-significant variables were selected for removal using the ‘drop1’ function. Models with the lowest Akaike information criterion (AIC) were retained. Juveniles were first excluded from full models as they were sampled during a short haul-out period that is not characterized by extensive fasting. In cases in which components did not vary with fasting in pups and adults, juveniles were replaced in the models to assess the effects of age and sex. Levene’s and Shapiro-Wilk’s tests were used to determine whether variables and model residuals met equal variance and normality assumptions, respectively. Variables were log-transformed as necessary to meet model assumptions. *Post hoc* comparisons between sample groups were conducted using estimated marginal means (EMM) with the *emmeans* package (adjustment = Tukey) (Lenth, 2021). GLM was also used to assess whether log-transformed CO levels varied by age, sex, and fasting. Spearman correlation was used to evaluate relationships between CO levels and rotated components.

## Results

### Carbon Monoxide Levels in Skeletal Muscle

**FIGURE 1:**
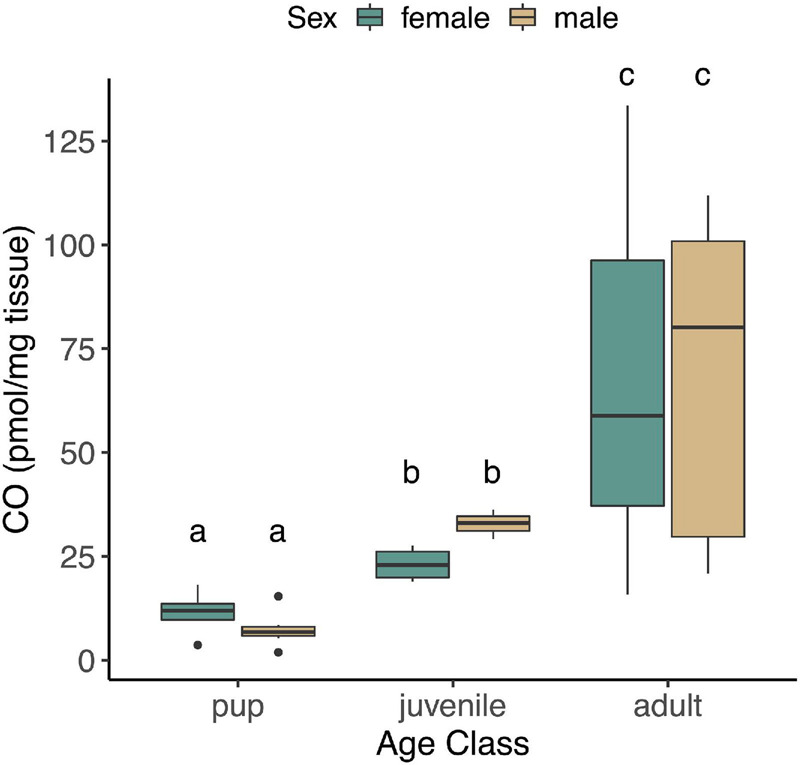
CO levels measured in skeletal muscle of NES pups (*n* = 13; 4 female, 9 male), juveniles (*n* = 7; 4 female, 3 male), and adults (*n* = 20; 10 female, 10 male). Different letters denote significant differences between groups (GLM using log-transformed CO, followed by *post hoc* EMM test, *p* < 0.05).

The concentration of CO in NES skeletal muscle was unaffected by fasting in pups or adults (*p* = 0.37), and none of the interaction terms that included fasting were significant (*p* > 0.05). These terms were therefore removed, and juveniles were included in reduced models with age, sex, and age^∗^sex. CO concentrations in skeletal muscle varied significantly with age (*F*_2,37_ = 43.15, *p* < 0.0001), but were unaffected by sex (*p* = 0.99, Figure 1) or the interaction between age and sex (*p* = 0.39). Skeletal muscle CO concentrations increased with age, from 8.5 ± 4.7 (mean ± S.D.) pmol^∗^mg^–1^ tissue in pups, to 26.7 ± 6.1 pmol^∗^mg^–1^ tissue in juveniles, and 67.1 ± 37.3 pmol^∗^mg^–1^ tissue in adults (Figure 1).

### Gene Expression in Skeletal Muscle

**FIGURE 2:**
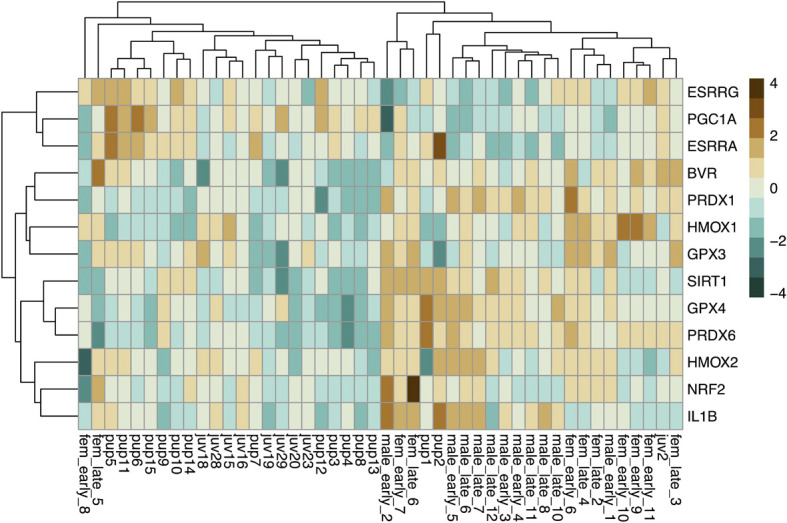
Heatmap showing scaled baseline expression (delta C_T_; higher: brown, lower: blue) of 13 genes (rows) associated with CO signaling and cytoprotection in skeletal muscle of NES pups (*n* = 15), juveniles (*n* = 9), and adults (*n* = 22; columns). Rows and columns were clustered by Euclidean distance.

**FIGURE 3:**
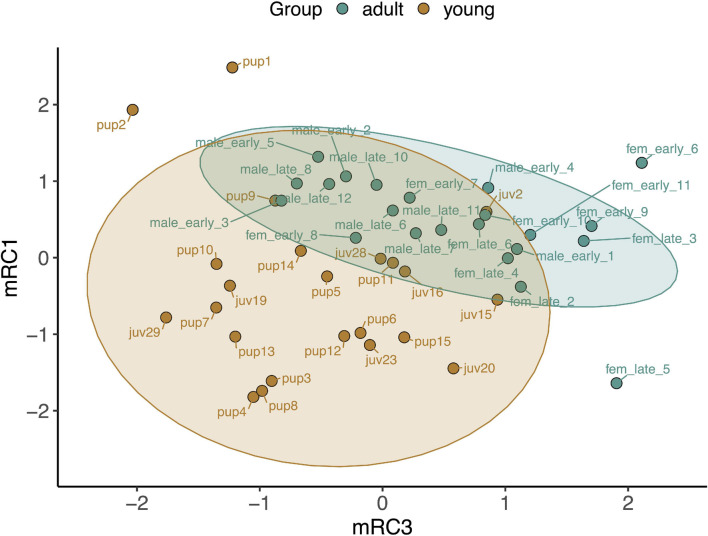
Principal components analysis loading plot for rotated components describing baseline gene expression associated with CO signaling and cytoprotection in skeletal muscle of NES pups, juveniles, and adults. mRC1: *GPX4*, *PRDX6*, *PRDX1*, *SIRT1*; mRC3: *HMOX1*, *BVR*, *GPX3*, *PRDX1*. Ellipses show 95% confidence intervals for young (pups and juveniles, *n* = 24; brown) and adult (*n* = 22; blue) seals.

Baseline gene expression in skeletal muscle was variable between individuals but was clustered by gene and age, with higher expression levels of most markers, with the exception of those related to mitochondrial biogenesis, in adults compared to pups and juveniles (Figure 2). We used PCA to reduce the dimensionality of the dataset as gene expression was highly correlated (Supplementary Figure 1). Muscle gene expression was described by four rotated components (Table 1), which in sum explained 77% of the variance in the data and showed clustering by age (young *vs.* adult, Figure 3).

**TABLE 1:** Eigenvalues and percent of explained variance for varimax-rotated components mRC1, mRC2, mRC3, and mRC4 that describe expression of genes associated with protection from oxidative stress and lipid peroxidation, production of CO, and mitochondrial biogenesis in skeletal muscle of NES.

	mRC1	mRC2	mRC3	mRC4
Eigenvalue	3.12	2.35	1.98	2.66
% of variance	24	18	15	20
Gene	Rotated component loadings			
*HMOX1*	0.12	−0.34	**0.80**	−0.07
*HMOX2*	0.07	0.00	0.12	**0.82**
*BVR*	0.33	0.40	**0.66**	0.16
*NRF2*	0.17	−0.01	0.20	**0.86**
*GPX3*	−0.16	−0.05	**0.66**	0.43
*GPX4*	**0.89**	−0.23	−0.04	0.16
*PRDX1*	**0.56**	−0.23	**0.51**	0.37
*PRDX6*	**0.92**	−0.16	0.22	0.01
*SIRT1*	**0.74**	0.03	0.02	0.42
*IL1B*	0.40	−0.09	−0.01	**0.70**
*PGC1A*	−0.49	**0.73**	−0.15	−0.08
*ESRRA*	−0.04	**0.88**	−0.24	0.19
*ESRRG*	−0.16	**0.78**	0.15	−0.40

*Rotated component loading scores are shown for each gene used in the analysis, with values >0.5 shown in bold.*

Muscle expression of *GPX4*, *PRDX1*, *PRDX6*, and *SIRT1*, which are associated with protection from lipid peroxidation, was described by rotated component 1 (mRC1) and explained 24% of the variance in the data. *PGC1A*, *ESRRA* and *ESRRG*, which are associated with mitochondrial biogenesis, loaded on rotated component 2 (mRC2; 18% of variance explained). Expression of *HMOX1*, *BVR*, *GPX3*, and *PRDX1*, which are associated with CO production and antioxidant function, was described by rotated component 3 (mRC3; 15% of variance explained). Lastly, *HMOX2*, *NRF2*, and *IL1B*, which are associated with responses to oxidative stress and inflammation, loaded on rotated component 4 (mRC4), which explained 20% of the variance in the data. *PRDX1*, which encodes an antioxidant enzyme, loaded on both mRC1 and mRC3.

**FIGURE 4:**
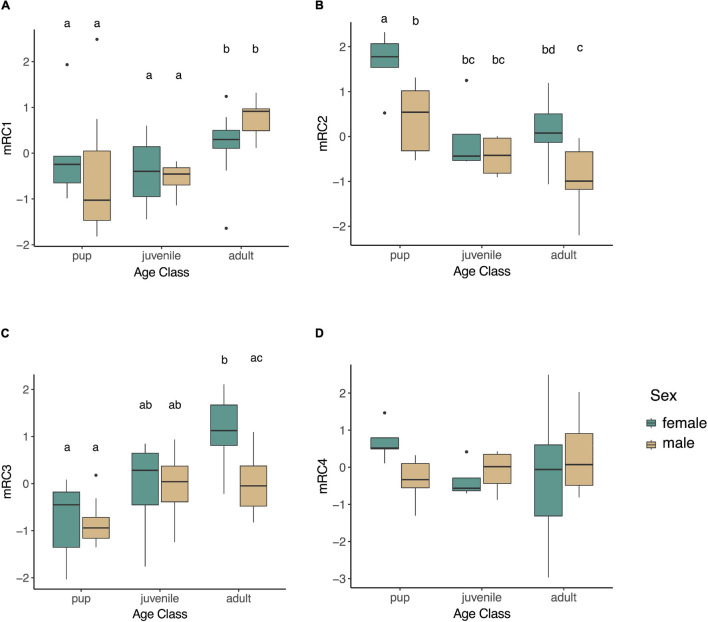
Boxplots showing expression of rotated components mRC1 (**A**; *GPX4*, *PRDX6*, *PRDX1*, *SIRT1*), mRC2 (**B**; *PGC1A*, *ESRRA*, *ESRRG*), mRC3 (**C**; *HMOX1*, *BVR*, *GPX3*, *PRDX1*), and mRC4 (**D**; *HMOX2*, *NRF2*, *IL1B*) in skeletal muscle of NES. mRC1 varied by age (*F*_2,42_ = 5.31, *p* = 0.0088), while mRC2 and mRC3 varied by age and sex (mRC2: *F*_4,41_ = 14.26, *p* < 0.0001; mRC3: *F*_2,39_ = 3.13, *p* = 0.055). Different letters denote significant differences between groups (GLM followed by *post hoc* EMM test, *p* < 0.05).

We examined whether any of the muscle-specific rotated components varied by age (pup, juvenile, adult), sex, or fasting state (early, late; in pups and adults only as juveniles were sampled during a haulout period not associated with prolonged fasting), or their interactions. None of the rotated components varied between animals sampled early and those that were sampled late in their fast in pups or adults (mRC1: *p* = 0.37, mRC2: *p* = 0.10, mRC3: *p* = 0.13, mRC4: *p* = 0.42), and none of the interaction terms that included fasting state were significant (*p* > 0.05). Therefore, these terms were removed, and juveniles were included in reduced models with age, sex, and age^∗^sex as explanatory variables. mRC1 expression increased with age (*F*_2,42_ = 5.31, *p* = 0.0088; Figure 4A); it was higher in adults compared to juveniles (*p* = 0.034) and pups (*p* = 0.026) but did not differ between pups and juveniles (*p* = 0.92). mRC1 did not vary by sex (*p* = 0.92) or the interaction between age and sex (*p* = 0.22). mRC2 expression decreased with age (*F*_2,41_ = 19.07, *p* < 0.0001; Figure 4B); it was higher in pups compared to juveniles (*p* = 0.016) and adults (*p* = 0.0002) but did not vary between adults and juveniles (*p* = 0.88). mRC2 expression was higher in females compared to males (*F*_1,41_ = 22.14, *p* < 0.0001), with highest levels in female pups and lowest levels in adult males (Figure 4B); the interaction term age^∗^sex was not significant (*p* = 0.37). mRC3 increased with age (*F*_2,39_ = 14.84, *p* < 0.0001). The interaction term age^∗^sex was marginally significant (*F*_2,39_ = 3.13, *p* = 0.055); mRC3 expression was higher in adult females compared to adult males (*p* = 0.0099; Figure 4C). mRC4 expression did not vary with age (*p* = 0.68), sex (*p* = 0.77), or their interaction (*p* = 0.088; Figure 4D).

**FIGURE 5:**
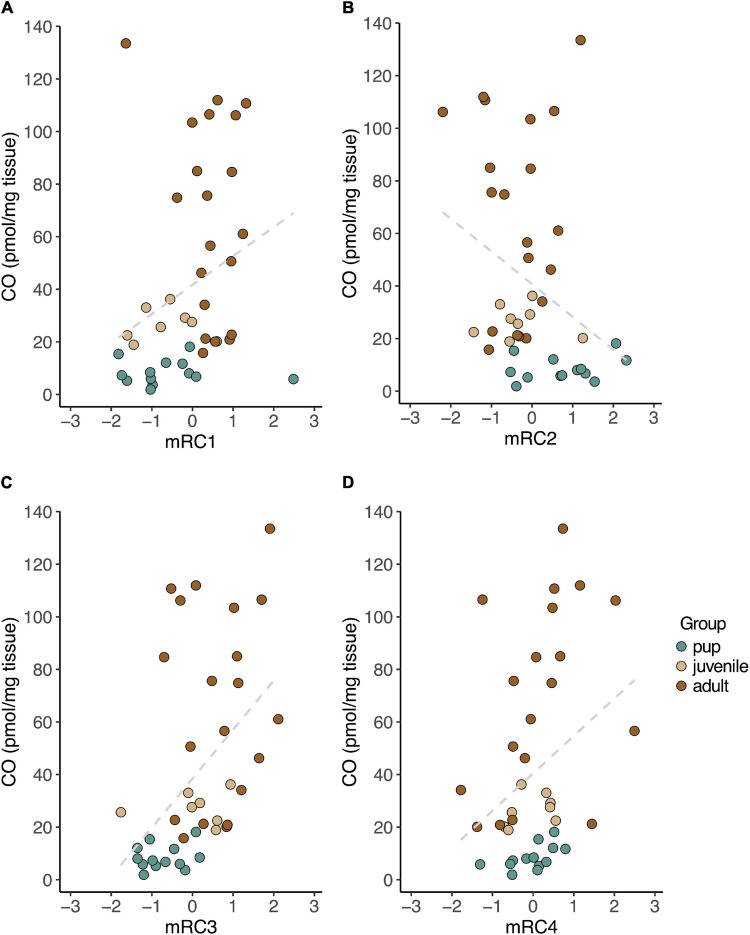
CO levels measured in skeletal muscle were positively correlated with baseline expression of mRC1 (**A**; *GPX4*, *PRDX6*, *PRDX1*, *SIRT1*; *r*_*S*_ = 0.43, *p* = 0.006), mRC3 (**C**; *HMOX1*, *BVR*, *GPX3*, *PRDX1*; *r*_*S*_ = 0.57, *p* = 0.0001), and mRC4 (**D**; *HMOX2*, *NRF2*, *IL1B*; *r*_*S*_ = 0.33, *p* = 0.034), and negatively correlated with expression of mRC2 (**B**; *PGC1A, ESRRA, ESRRG*; *r*_*S*_ = –0.36, *p* = 0.021) in NES.

We then evaluated relationships between CO levels and rotated component expression in muscle. Muscle CO levels were positively correlated with mRC1 (*r*_*S*_ = 0.43, *p* = 0.006; Figure 5A), mRC3 (*r*_*S*_ = 0.57, *p* = 0.0001; Figure 5C) and mRC4 (*r*_*S*_ = 0.33, *p* = 0.034; Figure 5D), and negatively correlated with mRC2 (*r*_*S*_ = −0.36, *p* = 0.021; Figure 5B).

### Gene Expression in Whole Blood

**FIGURE 6:**
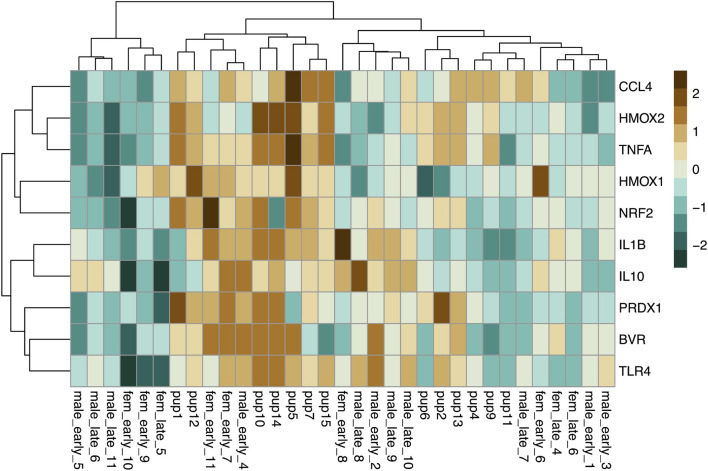
Heatmap showing scaled baseline expression (delta C_T_; higher: brown, lower: blue) of 10 genes (rows) associated with CO signaling, cytoprotection, and inflammation in whole blood of NES pups (*n* = 13) and adults (*n* = 20, columns). Rows and columns were clustered by Euclidean distance.

**FIGURE 7:**
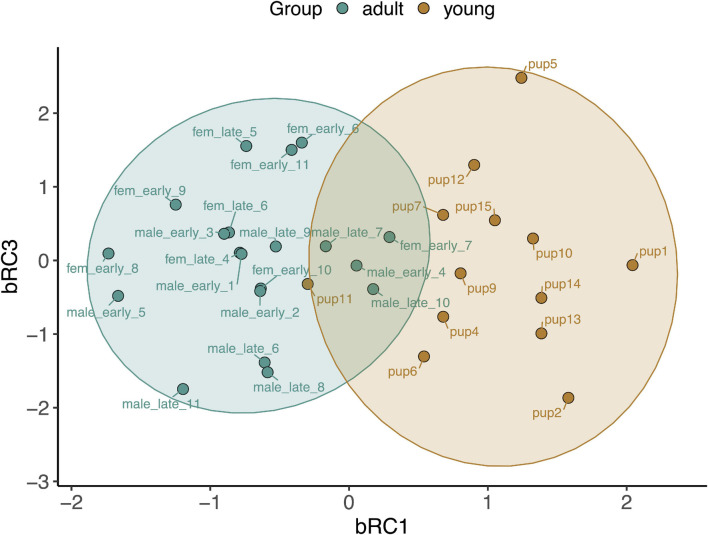
Principal components analysis loading plot for rotated components describing baseline gene expression associated with CO signaling, cytoprotection, and inflammation in whole blood of NES pups (*n* = 13) and adults (*n* = 20). bRC1: *HMOX2*, *PRDX1*, *TLR4*, *CCL4*, *TNFA*; bRC3: *HMOX1*. Ellipses show 95% confidence intervals for the two age classes.

We measured expression of *HMOX1*, *HMOX2*, *BVR*, *NRF2*, *PRDX1*, *IL1B* and four other markers associated with immune function, which were not detectable in muscle, in whole blood (*IL10*, *TLR4*, *TNFA*, *CCL4*). Baseline expression of these 10 genes in whole blood showed clustering by age, with higher expression levels of most markers, with the exception of *HMOX1* and *NRF2*, in pups compared to adults (Figure 6). Blood gene expression was described by three rotated components (Table 2), that in sum explained 79% of the variance in the data and showed clustering by age (young *vs.* adult, Figure 7). Expression of *HMOX2*, *PRDX1*, *CCL4*, and *TNFA* in whole blood, which are associated with antioxidant function and inflammation, loaded on rotated component 1 (bRC1) and explained 33% of the variance in the data. Expression of *IL1B*, *NRF2*, *BVR*, and *IL10*, which are associated with regulation of inflammation, was described by rotated component 2 (bRC2; 31% of variance explained). *HMOX1*, which is associated with CO production, loaded on rotated component 3 (bRC3; 15% of variance explained). *TLR4* loaded on both bRC1 and bRC2.

**TABLE 2:** Eigenvalues and percent of explained variance for varimax-rotated components bRC1, bRC2, and bRC3 that describe expression of genes associated with inflammation, protection from oxidative stress, and production of CO in whole blood of NES.

	bRC1	bRC2	bRC3
Eigenvalue	3.31	3.13	1.49
% of variance	33	31	15
Gene	Varimax-rotated component loadings		
*HMOX1*	0.28	0.10	**0.91**
*HMOX2*	**0.89**	0.07	0.27
*IL1B*	0.00	**0.85**	0.34
*NRF2*	0.43	**0.53**	0.45
*BVR*	0.36	**0.70**	0.22
*TLR4*	**0.51**	**0.77**	−0.06
*IL10*	0.01	**0.90**	−0.07
*CCL4*	**0.78**	0.09	0.22
*PRDX1*	**0.71**	0.43	−0.21
*TNFA*	**0.88**	0.19	0.34

*Rotated component loading scores are shown for each gene used in the analysis, with values >0.5 shown in bold.*

**FIGURE 8:**
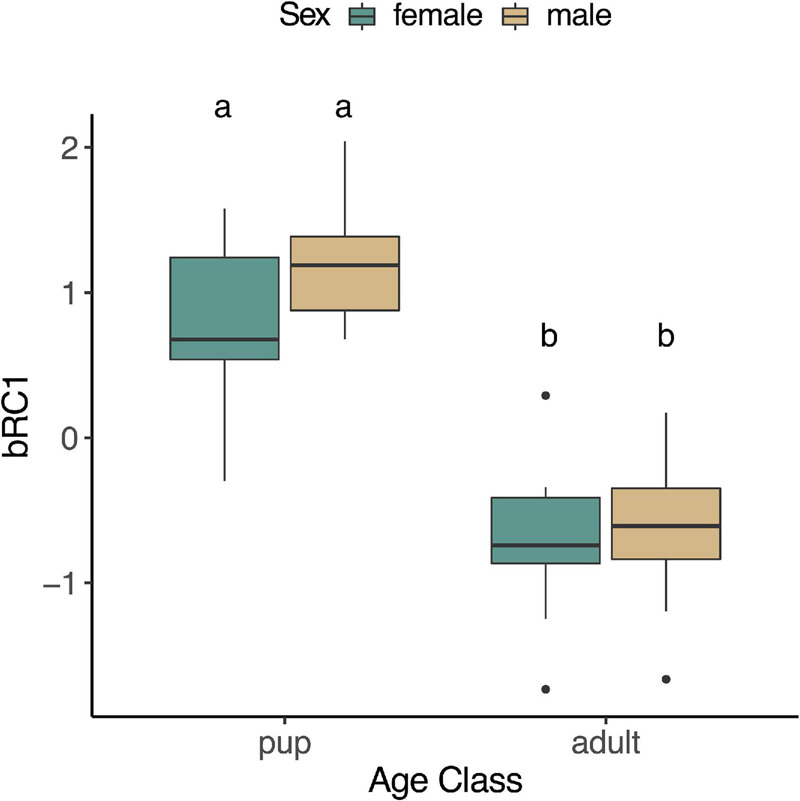
Expression of rotated component bRC1 (*HMOX2*, *PRDX1*, *TLR4*, *CCL4*, *TNFA*) in whole blood of NES was higher in pups than adults (*F*_*1,31*_ = 73.41, *p* < 0.05).

We examined whether any of the blood-specific rotated components varied by age, sex, fasting state, or their interaction in pups and adults. None of the components varied with fasting (bRC1: *p* = 0.14; bRC2: *p* = 0.48; bRC3: *p* = 0.78), and none of the interaction terms that included fasting were significant (*p* > 0.05); these terms were subsequently removed from the models. bRC1 expression decreased with age (*F*_1,31_ = 73.41, *p* < 0.0001; Figure 8), but was not affected by sex (*p* = 0.18) or the interaction between age and sex (*p* = 0.39). bRC2 and bRC3 did not vary by age (bRC2: *p* = 0.33; bRC3: *p* = 0.65), sex (bRC2: *p* = 0.32; bRC3: *p* = 0.12), or the interaction between age and sex (bRC2: *p* = 0.51; bRC3: *p* = 0.10; data not shown).

**FIGURE 9:**
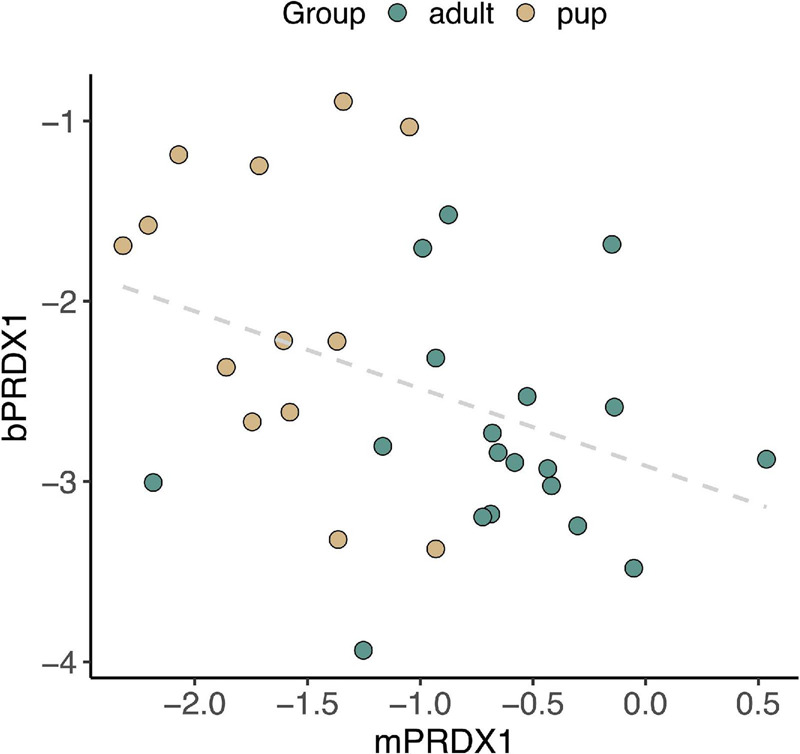
Expression of the gene encoding antioxidant enzyme PRDX1 in NES muscle (m*PRDX1*) and blood (b*PRDX1*) was negatively associated (*r_*S*_* = –0.40, *p* = 0.02).

Lastly, we examined potential associations between gene expression in skeletal muscle and peripheral blood. There was a significant inverse correlation between *PRDX1* expression in muscle and its expression in blood (*r_*S*_* = −0.40, *p* = 0.02; Figure 9). Expression levels of *HMOX1*, *HMOX2*, *BVR*, *IL1B*, and *NRF2* in skeletal muscle and blood were not correlated (*HMOX1*: *p* = 0.56, *HMOX2*: *p* = 0.31, *BVR*: *p* = 0.93, *IL1B*: *p* = 0.22, *NRF2*: *p* = 0.98).

## Discussion

Carbon monoxide has long been characterized as an anthropogenic pollutant and toxic gas due to its ability to significantly alter oxygen delivery in the body. Contrary to its former reputation, endogenously produced CO is now widely accepted as a gasotransmitter that confers cytoprotection in the face of hypoxemia, ischemia, and reperfusion in humans and laboratory species (Motterlini and Otterbein, 2010; Tift et al., 2020). However, the role and regulation of endogenous CO production and its potential to exert similar effects in species that are naturally adapted to chronic hypoxia have not been studied. One of the deepest-diving marine mammals, NES, produce and maintain CO at concentrations higher than those of cigarette smokers, and avoid tissue injuries typically seen in other mammals that experience repeated exposure to hypoxemia and ischemia-reperfusion events (Tift et al., 2014, 2020). Due to the large amount of evidence demonstrating the cytoprotective role of low to moderate CO exposure in humans and rodents (Motterlini and Otterbein, 2010), we hypothesized that the high levels of CO previously measured in NES may be related to expression of genes associated with regulation of oxidative stress and inflammatory pathways, thus potentially contributing to hypoxia tolerance in this deep-diving species.

Our previous work demonstrated that percent carboxyhemoglobin (COHb) levels increase across ontogeny in NES, from mean (±S.D.) 7.1 ± 0.3% in pups and 7.6 ± 0.2% in juveniles to 8.7 ± 0.3% in adults (Tift et al., 2014). Therefore, we hypothesized that tissue-level CO concentrations and expression of genes associated with CO production and cytoprotection would similarly increase as NES pups transitioned from a terrestrial lifestyle to elite diving adults. We found that the concentration of CO in skeletal muscle increased across ontogeny, as predicted from previous measurements of CO in the blood of NES (Tift et al., 2014). To our knowledge, these are the first measurements of endogenous tissue CO concentrations reported in a wild mammal. The mean concentrations of CO measured in skeletal muscle of NES pups (8.5 pmol/mg) resemble mean resting levels of CO in mice (10 pmol/mg). However, the mean (67.1 pmol/mg) and maximum (133.5 pmol/mg) CO concentrations seen in adult NES skeletal muscle are more similar to mean concentrations of CO found in skeletal muscle of humans that died during fires (102 pmol/mg), and much higher than mean CO concentrations in skeletal muscle of mice that inhaled 500 ppm CO for 30 min (14 pmol/mg) (Vreman et al., 2005, 2006). We believe that there are two possible explanations for such high CO concentrations in NES skeletal muscle. First, NES could be producing high quantities of CO directly in skeletal muscle, as a cytoprotective mechanism to alleviate potential injuries occurring in a tissue that experiences routine hypoxia and ischemia and reperfusion events. CO produced in skeletal muscle could directly be acting on other proteins (e.g., cGMP) to induce cytoprotective effects (Motterlini and Otterbein, 2010). We were unable to determine the subcellular localization of CO in NES skeletal muscle, but we suspect it is likely bound to a hemoprotein (e.g., myoglobin or cytochrome) based on the high affinity of CO for heme (Levitt and Levitt, 2015). NES pups and adults have approximately 10-fold and 17-fold higher myoglobin concentrations in skeletal muscle, respectively (Thorson and Le Boeuf, 1994; Hassrick et al., 2010), compared to humans (Möller and Sylvén, 1981). This represents a large *source* of heme for HO enzymes to produce endogenous CO. However, this quantity of myoglobin also represents a large *sink* to bind free CO delivered to the tissue, thus potentially inhibiting CO from binding to other critical hemoproteins (e.g., cytochrome-c-oxidase) (Almeida et al., 2015). A second potential explanation is that CO is being produced in other tissues (e.g., spleen), and is delivered to the skeletal muscle via blood. NES have the highest mass-specific blood volume of all mammals, with extremely high hemoglobin concentrations and hematocrit values (Hassrick et al., 2010). This represents another large heme store which could be used by HO enzymes to produce endogenous CO as product of erythrocyte turnover. We have already established that NES have a significant portion of their hemoglobin consistently bound to CO (Tift et al., 2014), yet it is not known how much of the gas is being transferred between blood and muscle. Diffusion of CO from blood into tissues may enable the gas to exert its potent cytoprotective effects, but it may also have deleterious impacts on oxygen delivery to the mitochondria. Considering that NES routinely dive for over an hour and spend very little time at the surface recovering from these long duration dives, suggesting minimal production of anaerobic metabolic byproducts during dives that must be dealt with at the surface (Thorson and Le Boeuf, 1994; Hassrick et al., 2010), we do not believe their onboard CO concentrations limit adequate oxygen delivery. Instead, we believe the high quantities of intravascular and extravascular CO seen in this species likely plays a cytoprotective role to resist injuries to tissues that routinely experience hypoxia and ischemia and reperfusion events (Tift et al., 2020).

We found that baseline gene expression in skeletal muscle was highly correlated by function, including (1) protection from lipid peroxidation (mRC1), (2) mitochondrial biogenesis (mRC2), (3) CO production (mRC3), and (4) regulation of inflammation (mRC4), all of which were associated with concentrations of CO in skeletal muscle. Gene expression in peripheral blood cells also clustered by function, including (1) protection from oxidative stress (bRC1), (2) regulation of inflammation (bRC2), and (3) CO production (bRC3, *HMOX1*). Of the genes evaluated in this study, none varied significantly with the duration of fasting that the animals experienced prior to sample collection. This was surprising due to the potential oxidative cost of fasting and previous reports of adaptive antioxidant responses in fasting NES (Ensminger et al., 2021). Our ability to detect differences in gene expression between early and late fasted seals may have been limited by a small sample size and warrants further investigation.

In skeletal muscle, expression of genes associated with protection from lipid peroxidation (mRC1: *GPX4*, *PRDX6*, *PRDX1*, *SIRT1*) was positively correlated with CO and increased with age, with highest expression in adult male NES. GPX4 and PRDX6 are antioxidant enzymes that can reduce peroxidized phospholipids and repair damaged cell membranes, protecting cells from ferroptosis, or death caused by lipid peroxidation (Fisher, 2017; Arevalo and Vázquez-Medina, 2018; Ursini and Maiorino, 2020). PRDX6 has also been linked to myogenesis and maintenance of muscle mass during aging in humans and mice (Pacifici et al., 2020; Soriano-Arroquia et al., 2021), suggesting a potentially similar role in adult NES. PRDX1, another member of the peroxiredoxin family, has a similar function as GPX4 and PRDX6, but relies on thioredoxin instead of glutathione as a reductant (Ding et al., 2017). Consistent with the positive correlation between CO and mRC1 genes in NES, CO administration and *PRDX6* overexpression in mice upregulate *SIRT1*, a key regulator of energy homeostasis and longevity that confers protection from lipid-induced inflammation (Pfluger et al., 2008; Kim et al., 2015; Pacifici et al., 2020). High expression of these genes is likely adaptive for animals such as NES that have large hemoprotein and lipid stores, high metabolic rates, and frequently experience ischemia/reperfusion events that can generate reactive oxygen species (ROS). It may also serve to compensate for the evolutionary loss of paraoxonase-1, a circulating antioxidant enzyme that protects lipids from oxidation, in marine mammals (Meyer et al., 2018). Compared to juvenile and adult female NES, adult males typically sustain longer diving-associated apneas at sea and higher fasting metabolic rates on land (Le Boeuf et al., 2000; Hassrick et al., 2007; Crocker et al., 2012). Due to their large body size, males also have larger hemoprotein stores, and thus have a higher potential for liberating the pro-oxidant iron during heme turnover, which also generates CO. Adult males, but not females, exhibit elevated levels of lipid peroxidation markers during prolonged fasts associated with breeding (Crocker et al., 2012; Sharick et al., 2015). Therefore, higher baseline expression of mRC1-associated genes in adult male NES may confer increased protection to animals that have greater risk for lipid peroxidation. Further work will be necessary to determine whether CO directly regulates *GPX4* and *PRDX6* expression in NES.

Muscle expression of genes involved in mitochondrial biogenesis (mRC2: *PGC1A*, *ESRRA*, *ESRRG*), which are upregulated by CO in other systems (Rhodes et al., 2009; Chan et al., 2016; Choi et al., 2016), was highest in NES pups and declined with age. This was despite age-related increases in *HMOX1* expression and CO levels in muscle and blood (Tift et al., 2014). PGC1A, a master regulator of mitochondrial biogenesis, enhances activity of the orphan estrogen-related receptors ESRRA and ESRRG, leading to increased endurance capacity and oxidative remodeling of tissues (Rangwala et al., 2010; Fan et al., 2018). In skeletal muscle, ESRRG is important for long-term exercise adaptation and ESRRA functions as a mediator of adaptive mitochondrial biogenesis, as it is co-expressed with *PGC1A* during development and under conditions of physiological stress (Villena et al., 2007; Giguère, 2008; Rangwala et al., 2010). High levels of *PGC1A*, *ESRRA*, and *ESRRG* expression may help prime rapidly developing NES for their first trip to sea, e.g., by stimulating the switch from glycolytic type II muscle fibers predominant in pups to aerobic type I muscle fibers characteristic of adults (Moore et al., 2014). Expression of mRC2 was higher in female compared to male NES, potentially due to the influence of sex hormones on mitochondrial bioenergetics (Sultanova et al., 2020). In humans, estrogen administered following trauma-induced hemorrhage increased *PGC1A* expression and mediated cardioprotection (Murphy and Steenbergen, 2007). Studies in other systems have shown that low doses of CO administration (up to 3% COHb) stimulate or activate mitochondrial biogenesis in skeletal muscle by increasing *PGC1A* mRNA expression (Rhodes et al., 2009). However, we found that mRC2 expression was inversely correlated with muscle CO levels and *HMOX1* expression in NES, which naturally experience CO levels of up to 9% COHb (Tift et al., 2014). It is possible that expression of genes associated with mitochondrial biogenesis is more responsive to rapidly increasing CO levels in pups during postnatal development than to sustained, high CO levels in adulthood. Alternatively, expression of these genes may be decoupled from the CO pathway in NES, potentially due to their unique metabolic adaptations to prolonged fasting and the role of PGC1A in promoting lipid oxidation in muscle (Gudiksen and Pilegaard, 2017). However, muscle *PGC1A* expression in NES muscle did not vary with fasting state in this or other studies (Wright et al., 2020), and its role in fasting and diving adaptations of NES requires further investigation.

Expression of genes associated with endogenous CO production (mRC3: *HMOX1*, *BVR*, *GPX3*, *PRDX1*) in muscle was positively correlated with the concentration of CO in skeletal muscle and increased with age, with highest expression detected in adult females. The breakdown of heme by HO enzymes is the primary source of endogenous CO and biliverdin, which significantly contribute to antioxidant and anti-inflammatory responses in animals (Jansen and Daiber, 2012; Canesin et al., 2020). BVR catalyzes the final step of the heme degradation pathway, converting biliverdin into the potent antioxidant bilirubin (Canesin et al., 2020). Expression of *HMOX1*, *BVR*, and *PRDX1* in other species is induced by intracellular free heme and can confer cytoprotection from injuries associated with hypoxia and ischemia-reperfusion events (Gozzelino et al., 2010). GPX3 is a selenium-dependent antioxidant enzyme that scavenges hydrogen peroxide, organic hydroperoxides, and peroxynitrite generated during normal metabolism or oxidative stress (Chang et al., 2020). It has been shown to confer cytoprotection on muscle cells exposed to a variety of stressors (Chung et al., 2009; El Haddad et al., 2012). Similar to *PGC1A*, *GPX3* expression is increased by estrogen (Baltgalvis et al., 2010). Age-related increases in expression of *GPX3* and *PRDX1* (as well as *GPX4* and *PRDX6*) in NES skeletal muscle is consistent with studies in other phocids showing that total GPx and Prx activity increases with age under basal conditions (Vázquez-Medina et al., 2011c). This suggests that increases in expression and activity of these antioxidant enzymes is a common feature of development of dive capacity in marine mammals. As adult female NES consistently perform dives above their calculated aerobic dive limit (Hassrick et al., 2010), potentially experiencing higher rates of oxygen depletion than adult males, elevated expression of genes associated with mRC3 may enable them to avoid oxidative damage to critical tissues by increasing or simply maintaining high quantities of endogenous CO and antioxidant enzymes. The expression of genes in mRC3 was positively correlated with concentration of CO in skeletal muscle, providing a potential link between *HMOX1* expression, CO production, and expression of cytoprotective factors, which may enable prolonged, deep diving in NES (Tift and Ponganis, 2019). Further functional studies will be necessary to test this hypothesis, as elevated CO levels in skeletal muscle of adults, compared to young NES, may simply be a consequence of elevated concentrations of hemoproteins (e.g., myoglobin), which could serve as a source for CO production via HO activity or a sink where CO could bind and be stored. Furthermore, a fraction of endogenous CO is derived from non-heme sources such as lipid peroxidation and the gut microbiome (Vreman et al., 2001), which warrant further investigation.

We found that muscle expression of genes associated with regulation of inflammation (mRC4: *HMOX2*, *NRF2*, *IL1B*) was positively correlated with CO levels but did not vary with age or sex. This result was consistent with reports of constitutive *HMOX2* expression in other systems (Ayer et al., 2016). HO-2 has been shown to play a critical role in inflammatory-reparative regulation, oxygen sensing, cytoprotection, and evolutionary adaptation to high altitude in humans (Seta et al., 2006; Simonson et al., 2010; Yang et al., 2016). Knockdown of *HMOX2* expression in mice aggravated corneal inflammatory damage and impaired angiogenesis and overall HO activity (Bellner et al., 2008). In laboratory rats, CO released by the pharmacological CO-releasing agent CORM-2 alleviated chronic inflammation in the gastric mucosa by accelerating healing and repairing mechanisms, which are intracellularly mediated by Nrf2 (Magierowska et al., 2019). Nrf2 activation, in response to ROS, leads to the transcription of genes, including *HMOX1*, that are involved in protection from oxidative stress induced by inflammation (Hennig et al., 2018). Administration of CO and overexpression or activation of Nrf2, HO-1, and HO-2 have been shown to inhibit IL-1β production in mouse studies (Muñoz-Sánchez and Chánez-Cárdenas, 2014; Kobayashi et al., 2016; Dugbartey, 2021). Oxidative stress-mediated activation of the Nod-like receptor protein 3 (NLRP3) inflammasome was also associated with increased expression of *NRF2*, *HMOX1*, and *IL1B* in humans with osteoarthritis (Chen et al., 2019). The positive correlation between CO levels and *NRF2*, *HMOX2*, and *IL1B* expression in NES muscle suggests that *NRF2* and *HMOX2* may be upregulated (and, consequently, CO produced) in conditions of high *IL1B* expression, potentially as an adaptive mechanism to ameliorate oxidative damage caused by inflammation. Alternatively, these genes may serve to regulate functions other than (or in addition to) regulation of inflammation in NES muscle. For instance, IL-1β has been shown to stimulate myoblast proliferation in response to muscle injury in mice (Otis et al., 2014) and to augment glucose uptake in skeletal muscle in response to exercise (Tsuchiya et al., 2018). Nrf2 has also been shown to reduce lipid accumulation and oxidative damage in mice with hepatic steatosis (Upadhyay et al., 2020). Recent studies in humans have linked HMOX2, Nrf2, and IL-1β with insulin resistance and obesity (Li et al., 2012, 2020; Crilly et al., 2016; Tan et al., 2018; Yao et al., 2020), which are two characteristics exhibited by fasting NES (Houser et al., 2013). Evidently, more research is needed to understand the link between CO and *IL1B* expression in diving, fasting-adapted mammals. Two caveats of our study include the measurement of mRNA levels, rather than cytokine protein secretion, and the measurement of gene expression under baseline, rather than inflammatory conditions. This makes it challenging to decipher the relationship between CO and inflammatory markers in this system. Ultimately, it would be interesting to see whether CO administration would decrease IL-1β production in seal cells in functional experiments.

Our study is the first to examine gene expression in whole blood of NES, which contains primarily circulating lymphocytes and monocytes (PBMCs) (He et al., 2019). We hypothesized that exposure of PBMCs to fluctuating oxygen tension during apneas in NES would stimulate adaptive responses in this diving-adapted species (Stockard et al., 2007; Vázquez-Medina et al., 2011a; Tift et al., 2013), upregulating *HMOX1* expression and local CO production and regulating expression of pro-inflammatory cytokines. Due to the potent anti-inflammatory effects of CO reported in laboratory species, we predicted that *HMOX1* expression would be negatively correlated with pro-inflammatory markers and positively correlated with anti-inflammatory cytokines. In a previous study, exposure of mice to low CO concentrations under inflammatory conditions inhibited production of TNF-α, MIP-1β, and IL-1β and induced expression of *IL10* via a p38 MAPK-dependent mechanism (Ryter, 2020). CO also significantly suppressed lipopolysaccharide (LPS)-induced NADPH oxidase-dependent ROS generation in mouse macrophages by inhibiting *TLR4* and its downstream signaling pathways (Nakahira et al., 2006). Contrary to our predictions, *HMOX1* expression in blood did not vary by age and loaded onto a separate component (bRC3) that was not associated with any inflammatory markers. These data suggest that baseline *HMOX1* expression in PBMCs may be low and potentially decoupled from regulation of the markers tested in this study in a hypoxia-tolerant mammal. Baseline variability in *HMOX1* expression may also not reflect HO-1 enzyme abundance, activity, and role in immune, redox, and metabolic homeostasis under conditions of hypoxia-related inflammation or injury, which are rarely experienced by marine mammals (Allen and Vázquez-Medina, 2019). Further work will be necessary to elucidate the effects of CO on inflammatory signaling in marine mammals, especially since a recent study suggested that serum from NES and Weddell seals possessed intrinsic anti-inflammatory properties, the source of which has not yet been identified (Bagchi et al., 2018).

*HMOX2* expression in whole blood of NES was positively correlated with three pro-inflammatory cytokines (*TLR4*, *CCL4*, *TNFA*) and the antioxidant *PRDX1* (bRC1). Their expression was higher in pups compared to adults, despite the fact that older animals dive longer and deeper than juveniles and experience significant blood O_2_ depletion during routine dives (Meir et al., 2013), a condition that would trigger inflammation in humans or rodents (Allen and Vázquez-Medina, 2019). Higher bRC1 expression in young NES may reflect preconditioning responses to diving during postnatal development. During the post-weaning period, NES pups rapidly increase the duration spent submerged in shallow water along with the duration of sleep apneas on land, increasing their exposure to hypoxia (Blackwell and Boeuf, 1993). Repeated apneas in NES pups have been shown to potentiate mechanisms associated with protection from oxidative stress, including upregulation of hypoxia inducible factors (*HIF*s) (Vázquez-Medina et al., 2011a). The correlation between *TLR4* and *PRDX1* expression in NES blood is consistent with studies in mice that have shown that PRDX1, which is upregulated in response to ischemia-reperfusion, serves as an endogenous ligand for TLR4 (Liu and Zhang, 2019). Furthermore, the interaction between PRDX1 and TLR4 in human cancer cells was shown to upregulate HIF-1α (Riddell et al., 2012), a master regulator of adaptive responses to hypoxia that is highly expressed in NES tissues (Allen and Vázquez-Medina, 2019). The negative correlation between *PRDX1* expression in skeletal muscle and blood observed in this study highlights its complex, cell type-dependent functions in animals (Hopkins and Neumann, 2019), e.g., regulation of inflammatory signaling in PBMCs and lipid peroxidation in skeletal muscle. Our data suggest that postnatal development in a deep-diving mammal may involve priming the immune system by upregulating the oxygen sensor *HMOX2* and inflammatory markers that induce adaptive responses to hypoxia.

Lastly, we found that expression of anti-inflammatory and antioxidant markers (*IL10*, *NRF2*, *BVR*) in NES blood was positively associated with expression of pro-inflammatory markers (*IL1B*, *TLR4*). While this may seem paradoxical, recent studies have suggested that relationships between pro- and anti-inflammatory responses in mammals are extremely complex (Kowsar et al., 2019). For instance, *IL10* and *IL1B* are co-expressed under pathophysiological and physiological conditions in bovine cells (Kowsar et al., 2019), and IL-10 was recently shown to possess pro-inflammatory properties (Mühl, 2013). While primarily considered a pro-inflammatory marker, IL-1β also influences insulin secretion and insulin resistance in mice (Dror et al., 2017), and could therefore play a primarily metabolic role in fasting-adapted NES, which display insulin resistance (Champagne et al., 2012). The correlation between *BVR* and *IL10* expression in NES blood is consistent with data from other studies showing that BVR upregulates *IL10* by activating PI3K-Akt (Wegiel and Otterbein, 2012). However, BVR also directly inhibits *TLR4* expression (Medzhitov, 2001; Wegiel and Otterbein, 2012), while Nrf2 is known to suppress *IL1B* (Campbell et al., 2021). Co-expression of these factors in NES blood suggests a complex interplay of hormetic responses in a hypoxia-adapted mammal that warrant further mechanistic investigation.

## Conclusion

Our study is the first to measure tissue CO levels in any wild animal, and to report expression of genes associated with endogenous CO production and signaling in blood and muscle of a deep-diving phocid species across ontogeny. As such, it provides a number of hypotheses for further exploration of natural hypoxia tolerance in mammals. We propose that upregulation of baseline *HMOX1* expression in skeletal muscle of NES may, in part, underlie developmental increases in CO levels and expression of genes encoding cytoprotective factors such as antioxidant enzymes, several of which are involved in protection from lipid peroxidation. *HMOX2* may play a role in regulating inflammation related to ischemia and reperfusion in muscle and PBMCs of NES. Our data propose putative ontogenetic mechanisms that may enable phocid pups to transition to a deep-diving lifestyle. These include high expression of genes associated with mitochondrial biogenesis in muscle and potential immune system activation during postnatal development and age-related increases in expression of genes associated with protection from lipid peroxidation in adulthood. Functional studies, such as *in vitro* manipulations of CO levels and HO expression and activity will be necessary to determine the nature of the relationship between the CO/HO pathway and cytoprotective factors in diving mammals.

## Data Availability Statement

The datasets generated for this study can be found at figshare: https://figshare.com/s/91c6c1d1d230bf7a16e0.

## Ethics Statement

The animal study was reviewed and approved by Sonoma State University and University of the Pacific Institutional Animal Care and Use Committees and conducted under National Marine Fisheries Service permit Nos. 19108 and 23188.

## Author Contributions

MT, JK, and DC conceived and designed the study. EP, JK, DC, and AK collected the animal samples. EP, JK, and JV-M designed the gene expression assays. EP conducted the gene expression analyses. AP and MT measured carbon monoxide. JV-M, MT, and DC aided in interpreting results. EP and JK conducted statistical analyses and drafted the manuscript. All authors reviewed and approved the final version of the manuscript.

## Conflict of Interest

The authors declare that the research was conducted in the absence of any commercial or financial relationships that could be construed as a potential conflict of interest. The handling editor declared a past co-authorship with one of the author MT.

## Publisher’s Note

All claims expressed in this article are solely those of the authors and do not necessarily represent those of their affiliated organizations, or those of the publisher, the editors and the reviewers. Any product that may be evaluated in this article, or claim that may be made by its manufacturer, is not guaranteed or endorsed by the publisher.

## References

[B1] Allen K. N. Vázquez-Medina J. P. (2019). Natural tolerance to ischemia and hypoxemia in diving mammals: a review. *Front. Physiol.* 10:1199. 10.3389/fphys.2019.01199 31620019 PMC6763568

[B2] Almeida A. S. Figueiredo-Pereira C. Vieira H. L. A. (2015). Carbon monoxide and mitochondria—modulation of cell metabolism, redox response and cell death. *Front. Physiol.* 6:33. 10.3389/fphys.2015.00033 25709582 PMC4321562

[B3] Andrews R. D. Jones D. R. Williams J. D. Thorson P. H. Oliver G. W. Costa D. P. (1997). Heart rates of northern elephant seals diving at sea and resting on the beach. *J. Exp. Biol.* 200 2083–2095. 10.1242/jeb.200.15.2083 9255950

[B4] Arevalo J. A. Vázquez-Medina J. P. (2018). The role of peroxiredoxin 6 in cell signaling. *Antioxidants* 7:172. 10.3390/antiox7120172 30477202 PMC6316032

[B5] Ayer A. Zarjou A. Agarwal A. Stocker R. (2016). Heme oxygenases in cardiovascular health and disease. *Physiol. Rev.* 96 1449–1508. 10.1152/physrev.00003.2016 27604527 PMC5504454

[B6] Bagchi A. Batten A. J. Levin M. Allen K. N. Fitzgerald M. L. Huckstadt L. A. (2018). Intrinsic anti-inflammatory properties in the serum of two species of deep-diving seal. *J. Exp. Biol.* 221(Pt 13):jeb178491. 10.1242/jeb.178491 29748216

[B7] Baltgalvis K. A. Greising S. M. Warren G. L. Lowe D. A. (2010). Estrogen regulates estrogen receptors and antioxidant gene expression in mouse skeletal muscle. *PLoS One* 5:e10164. 10.1371/journal.pone.0010164 20405008 PMC2854140

[B8] Bellner L. Vitto M. Patil K. A. Dunn M. W. Regan R. Laniado-Schwartzman M. (2008). Exacerbated corneal inflammation and neovascularization in the HO-2 null mice is ameliorated by biliverdin. *Exp. Eye Res.* 87 268–278. 10.1016/j.exer.2008.06.007 18602389 PMC2628556

[B9] Blackwell S. B. Boeuf B. J. L. (1993). Developmental aspects of sleep apnoea in northern elephant seals, *Mirounga angustirostris*. *J. Zool.* 231 437–447. 10.1111/j.1469-7998.1993.tb01930.x

[B10] Campbell N. K. Fitzgerald H. K. Dunne A. (2021). Regulation of inflammation by the antioxidant haem oxygenase 1. *Nat. Rev. Immunol.* 21 411–425. 10.1038/s41577-020-00491-x 33514947

[B11] Canesin G. Hejazi S. M. Swanson K. D. Wegiel B. (2020). Heme-derived metabolic signals dictate immune responses. *Front. Immunol.* 11:66. 10.3389/fimmu.2020.00066 32082323 PMC7005208

[B12] Champagne C. D. Crocker D. E. Fowler M. A. Houser D. S. (2012). “Fasting physiology of the pinnipeds: the challenges of fasting while maintaining high energy expenditure and nutrient delivery for lactation,” in *Comparative Physiology of Fasting, Starvation, and Food Limitation*, ed. McCue M. D. (Berlin: Springer), 309–336.

[B13] Chan M. C. Ziegler O. Liu L. Rowe G. C. Das S. Otterbein L. E. (2016). Heme oxygenase and carbon monoxide protect from muscle dystrophy. *Skelet. Muscle* 6:41. 10.1186/s13395-016-0114-6 27906108 PMC5126804

[B14] Chang C. Worley B. L. Phaëton R. Hempel N. (2020). Extracellular glutathione peroxidase GPx3 and its role in cancer. *Cancers* 12:2197. 10.3390/cancers12082197 32781581 PMC7464599

[B15] Chen W. Maghzal G. J. Ayer A. Suarna C. Dunn L. L. Stocker R. (2018). Absence of the biliverdin reductase-a gene is associated with increased endogenous oxidative stress. *Free Radic. Biol. Med.* 115 156–165. 10.1016/j.freeradbiomed.2017.11.020 29195835

[B16] Chen Z. Zhong H. Wei J. Lin S. Zong Z. Gong F. (2019). Inhibition of Nrf2/HO-1 signaling leads to increased activation of the NLRP3 inflammasome in osteoarthritis. *Arthritis Res. Ther.* 21:300. 10.1186/s13075-019-2085-6 31870428 PMC6929452

[B17] Choi Y. K. Park J. H. Baek Y.-Y. Won M.-H. Jeoung D. Lee H. (2016). Carbon monoxide stimulates astrocytic mitochondrial biogenesis via L-type Ca2+ channel-mediated PGC-1α/ERRα activation. *Biochem. Biophys. Res. Commun.* 479 297–304. 10.1016/j.bbrc.2016.09.063 27639646

[B18] Chung S. S. Kim M. Youn B.-S. Lee N. S. Park J. W. Lee I. K. (2009). Glutathione peroxidase 3 mediates the antioxidant effect of peroxisome proliferator-activated receptor γ in human skeletal muscle cells. *Mol. Cell. Biol.* 29 20–30. 10.1128/MCB.00544-08 18936159 PMC2612482

[B19] Crilly M. J. Tryon L. D. Erlich A. T. Hood D. A. (2016). The role of Nrf2 in skeletal muscle contractile and mitochondrial function. *J. Appl. Physiol.* 121 730–740. 10.1152/japplphysiol.00042.2016 27471236 PMC5142253

[B20] Crocker D. E. Houser D. S. Webb P. M. (2012). Impact of body reserves on energy expenditure, water flux, and mating success in breeding male northern elephant seals. *Physiol. Biochem. Zool.* 85 11–20. 10.1086/663634 22237285

[B21] Deyarmin J. S. McCormley M. C. Champagne C. D. Stephan A. P. Busqueta L. P. Crocker D. E. (2019). Blubber transcriptome responses to repeated ACTH administration in a marine mammal. *Sci. Rep.* 9:2718. 10.1038/s41598-019-39089-2 30804370 PMC6390094

[B22] Ding C. Fan X. Wu G. (2017). Peroxiredoxin 1 – an antioxidant enzyme in cancer. *J. Cell. Mol. Med.* 21 193–202. 10.1111/jcmm.12955 27653015 PMC5192802

[B23] Dolar M. L. Suarez P. Ponganis P. J. Kooyman G. L. (1999). Myoglobin in pelagic small cetaceans. *J. Exp. Biol.* 202 227–236. 10.1242/jeb.202.3.227 9882635

[B24] Dror E. Dalmas E. Meier D. T. Wueest S. Thévenet J. Thienel C. (2017). Postprandial macrophage-derived IL-1β stimulates insulin, and both synergistically promote glucose disposal and inflammation. *Nat. Immunol.* 18 283–292. 10.1038/ni.3659 28092375

[B25] Dugbartey G. J. (2021). Carbon monoxide as an emerging pharmacological tool to improve lung and liver transplantation protocols. *Biochem. Pharmacol.* 193:114752. 10.1016/j.bcp.2021.114752 34487717

[B26] El Haddad M. Jean E. Turki A. Hugon G. Vernus B. Bonnieu A. (2012). Glutathione peroxidase 3, a new retinoid target gene, is crucial for human skeletal muscle precursor cell survival. *J. Cell Sci.* 125(Pt 24) 6147–6156. 10.1242/jcs.115220 23132926

[B27] Elsner R. Øyasæter S. Almaas R. Saugstad O. D. (1998). Diving seals, ischemia-reperfusion and oxygen radicals. *Comp. Biochem. Physiol. A Mol. Integr. Physiol.* 119 975–980. 10.1016/S1095-6433(98)00012-9 9773490

[B28] Ensminger D. C. Salvador-Pascual A. Arango B. G. Allen K. N. Vázquez-Medina J. P. (2021). Fasting ameliorates oxidative stress: a review of physiological strategies across life history events in wild vertebrates. *Comp. Biochem. Physiol. A Mol. Integr. Physiol.* 256:110929. 10.1016/j.cbpa.2021.110929 33647461

[B29] Fan W. He N. Lin C. S. Wei Z. Hah N. Waizenegger W. (2018). ERRγ promotes angiogenesis, mitochondrial biogenesis, and oxidative remodeling in PGC1α/β-deficient muscle. *Cell Rep.* 22 2521–2529. 10.1016/j.celrep.2018.02.047 29514081 PMC5860878

[B30] Field A. Miles J. Field Z. (2012). *Discovering Statistics Using R.* London: SAGE Publishing Ltd.

[B31] Fisher A. B. (2017). Peroxiredoxin 6 in the repair of peroxidized cell membranes and cell signaling. *Arch. Biochem. Biophys.* 617 68–83. 10.1016/j.abb.2016.12.003 27932289 PMC5810417

[B32] Giguère V. (2008). Transcriptional control of energy homeostasis by the estrogen-related receptors. *Endocr. Rev.* 29 677–696. 10.1210/er.2008-0017 18664618

[B33] Gozzelino R. Jeney V. Soares M. P. (2010). Mechanisms of cell protection by heme oxygenase-1. *Annu. Rev. Pharmacol. Toxicol.* 50 323–354. 10.1146/annurev.pharmtox.010909.105600 20055707

[B34] Gudiksen A. Pilegaard H. (2017). PGC-1α and fasting-induced PDH regulation in mouse skeletal muscle. *Physiol. Rep.* 5:e13222. 10.14814/phy2.13222 28400503 PMC5392513

[B35] Hassrick J. L. Crocker D. E. Teutschel N. M. McDonald B. I. Robinson P. W. Simmons S. E. (2010). Condition and mass impact oxygen stores and dive duration in adult female northern elephant seals. *J. Exp. Biol.* 213 585–592. 10.1242/jeb.037168 20118309

[B36] Hassrick J. L. Crocker D. E. Zeno R. L. Blackwell S. B. Costa D. P. Le Boeuf B. J. (2007). Swimming speed and foraging strategies of northern elephant seals. *Deep Sea Res. II Top. Stud. Oceanogr.* 54 369–383. 10.1016/j.dsr2.2006.12.001

[B37] He D. Yang C. X. Sahin B. Singh A. Shannon C. P. Oliveria J.-P. (2019). Whole blood vs PBMC: compartmental differences in gene expression profiling exemplified in asthma. *Allergy Asthma Clin. Immunol.* 15:67. 10.1186/s13223-019-0382-x 31832069 PMC6873413

[B38] Hennig P. Garstkiewicz M. Grossi S. Di Filippo M. French L. E. Beer H.-D. (2018). The crosstalk between Nrf2 and inflammasomes. *Int. J Mol. Sci.* 19:562. 10.3390/ijms19020562 29438305 PMC5855784

[B39] Hopkins B. L. Neumann C. A. (2019). Redoxins as gatekeepers of the transcriptional oxidative stress response. *Redox Biol.* 21:101104. 10.1016/j.redox.2019.101104 30690320 PMC6351230

[B40] Horning M. Trillmich F. (1997). Development of hemoglobin, hematocrit, and erythrocyte values in Galápagos fur seals. *Mar. Mamm. Sci.* 13 100–113. 10.1111/j.1748-7692.1997.tb00614.x

[B41] Houser D. S. Champagne C. D. Crocker D. E. (2013). A non-traditional model of the metabolic syndrome: the adaptive significance of insulin resistance in fasting-adapted seals. *Front. Endocrinol.* 4:164. 10.3389/fendo.2013.00164 24198811 PMC3814516

[B42] Jansen T. Daiber A. (2012). Direct antioxidant properties of bilirubin and biliverdin. Is there a role for biliverdin reductase? *Front. Pharmacol.* 3:30. 10.3389/fphar.2012.00030 22438843 PMC3306014

[B43] Jelincic J. A. Tift M. S. Houser D. S. Crocker D. E. (2017). Variation in adrenal and thyroid hormones with life-history stage in juvenile northern elephant seals (*Mirounga angustirostris*). *Gen. Comp. Endocrinol.* 252 111–118. 10.1016/j.ygcen.2017.08.001 28782534

[B44] Khudyakov J. I. Champagne C. D. Preeyanon L. Ortiz R. M. Crocker D. E. (2015). Muscle transcriptome response to ACTH administration in a free-ranging marine mammal. *Physiol. Genomics* 47 318–330. 10.1152/physiolgenomics.00030.2015 26038394 PMC4525076

[B45] Kim H. J. Joe Y. Yu J. K. Chen Y. Jeong S. O. Mani N. (2015). Carbon monoxide protects against hepatic ischemia/reperfusion injury by modulating the miR-34a/SIRT1 pathway. *Biochim. Biophys. Acta Mol. Basis Dis.* 1852 1550–1559. 10.1016/j.bbadis.2015.04.017 25916635

[B46] Kobayashi E. H. Suzuki T. Funayama R. Nagashima T. Hayashi M. Sekine H. (2016). Nrf2 suppresses macrophage inflammatory response by blocking proinflammatory cytokine transcription. *Nat. Commun.* 7:11624. 10.1038/ncomms11624 27211851 PMC4879264

[B47] Kolde R. (2019). *pheatmap: Pretty Heatmaps. R Package Version 1.0.12.* Available online at: https://cran.r-project.org/web/packages/pheatmap

[B48] Kooyman G. L. Ponganis P. J. (1998). The physiological basis of diving to depth: birds and mammals. *Annu. Rev. Physiol.* 60 19–32. 10.1146/annurev.physiol.60.1.19 9558452

[B49] Kowsar R. Keshtegar B. Miyamoto A. (2019). Understanding the hidden relations between pro- and anti-inflammatory cytokine genes in bovine oviduct epithelium using a multilayer response surface method. *Sci. Rep.* 9:3189. 10.1038/s41598-019-39081-w 30816156 PMC6395797

[B50] Le Boeuf B. J. Crocker D. E. Costa D. P. Blackwell S. B. Webb P. M. Houser D. S. (2000). Foraging ecology of northern elephant seals. *Ecol. Monogr.* 70 353–382.

[B51] Lenth R. V. (2021). *emmeans: Estimated Marginal Means, aka Least-Squares Means. R Package Version 1.6.1.* Available online at: https://cran.r-project.org/web/packages/emmeans

[B52] Levitt D. G. Levitt M. D. (2015). Carbon monoxide: a critical quantitative analysis and review of the extent and limitations of its second messenger function. *Clin. Pharmacol. Adv. Appl.* 7 37–56. 10.2147/cpaa.S79626 25750547 PMC4348054

[B53] Leys C. Klein O. Dominicy Y. Ley C. (2018). Detecting multivariate outliers: use a robust variant of the Mahalanobis distance. *J. Exp. Soc. Psychol.* 74 150–156. 10.1016/j.jesp.2017.09.011

[B54] Li B. Takeda K. Ishikawa K. Yoshizawa M. Sato M. Shibahara S. (2012). Coordinated expression of 6-phosphofructo-2-kinase/fructose-2,6-bisphosphatase 4 and heme oxygenase 2: evidence for a regulatory link between glycolysis and heme catabolism. *Tohoku J. Exp. Med.* 228 27–41. 10.1620/tjem.228.27 22892400

[B55] Li S. Eguchi N. Lau H. Ichii H. (2020). The role of the Nrf2 signaling in obesity and insulin resistance. *Int. J. Mol. Sci.* 21:6973. 10.3390/ijms21186973 32971975 PMC7555440

[B56] Lin Q. Weis S. Yang G. Weng Y.-H. Helston R. Rish K. (2007). Heme oxygenase-1 protein localizes to the nucleus and activates transcription factors important in oxidative stress. *J. Biol. Chem.* 282 20621–20633. 10.1074/jbc.M607954200 17430897

[B57] Liu Q. Zhang Y. (2019). PRDX1 enhances cerebral ischemia-reperfusion injury through activation of TLR4-regulated inflammation and apoptosis. *Biochem. Biophys. Res. Commun.* 519 453–461. 10.1016/j.bbrc.2019.08.077 31526567

[B58] Magierowska K. Bakalarz D. Wójcik D. Chmura A. Hubalewska-Mazgaj M. Licholai S. (2019). Time-dependent course of gastric ulcer healing and molecular markers profile modulated by increased gastric mucosal content of carbon monoxide released from its pharmacological donor. *Biochem. Pharmacol.* 163 71–83. 10.1016/j.bcp.2019.02.011 30753813

[B59] Medzhitov R. (2001). Toll-like receptors and innate immunity. *Nat. Rev. Immunol.* 1 135–145. 10.1038/35100529 11905821

[B60] Meir J. U. Champagne C. D. Costa D. P. Williams C. L. Ponganis P. J. (2009). Extreme hypoxemic tolerance and blood oxygen depletion in diving elephant seals. *Am. J. Physiol. Regul. Integr. Comp. Physiol.* 297 R927–R939. 10.1152/ajpregu.00247.2009 19641132

[B61] Meir J. U. Robinson P. W. Vilchis L. I. Kooyman G. L. Costa D. P. Ponganis P. J. (2013). Blood oxygen depletion is independent of dive function in a deep diving vertebrate, the northern elephant seal. *PLoS One* 8:e83248. 10.1371/journal.pone.0083248 24376671 PMC3871621

[B62] Meyer W. K. Jamison J. Richter R. Woods S. E. Partha R. Kowalczyk A. (2018). Ancient convergent losses of paraoxonase 1 yield potential risks for modern marine mammals. *Science* 361 591–594. 10.1126/science.aap7714 30093596 PMC6317340

[B63] Möller P. Sylvén C. (1981). Myoglobin in human skeletal muscle. *Scand. J. Clin. Lab. Invest.* 41 479–482. 10.3109/00365518109090486 7313529

[B64] Moore C. D. Crocker D. E. Fahlman A. Moore M. J. Willoughby D. S. Robbins K. A. (2014). Ontogenetic changes in skeletal muscle fiber type, fiber diameter and myoglobin concentration in the Northern elephant seal (*Mirounga angustirostris*). *Front. Physiol.* 5:217. 10.3389/fphys.2014.00217 24959151 PMC4050301

[B65] Motterlini R. Otterbein L. E. (2010). The therapeutic potential of carbon monoxide. *Nat. Rev. Drug Discov.* 9 728–743. 10.1038/nrd3228 20811383

[B66] Mühl H. (2013). Pro-Inflammatory signaling by IL-10 and IL-22: bad habit stirred up by interferons? *Front. Immunol.* 4:18. 10.3389/fimmu.2013.00018 23382730 PMC3562761

[B67] Muñoz-Sánchez J. Chánez-Cárdenas M. E. (2014). A review on hemeoxygenase-2: focus on cellular protection and oxygen response. *Oxid. Med. Cell. Longev.* 2014:604981. 10.1155/2014/604981 25136403 PMC4127239

[B68] Murphy E. Steenbergen C. (2007). Gender-based differences in mechanisms of protection in myocardial ischemia–reperfusion injury. *Cardiovasc. Res.* 75 478–486. 10.1016/j.cardiores.2007.03.025 17466956

[B69] Nakahira K. Kim H. P. Geng X. H. Nakao A. Wang X. Murase N. (2006). Carbon monoxide differentially inhibits TLR signaling pathways by regulating ROS-induced trafficking of TLRs to lipid rafts. *J. Exp. Med.* 203 2377–2389. 10.1084/jem.20060845 17000866 PMC2118097

[B70] Otis J. S. Niccoli S. Hawdon N. Sarvas J. L. Frye M. A. Chicco A. J. (2014). Pro-inflammatory mediation of myoblast proliferation. *PLoS One* 9:e92363. 10.1371/journal.pone.0092363 24647690 PMC3960233

[B71] Otterbein L. E. Bach F. H. Alam J. Soares M. Tao Lu H. Wysk M. (2000). Carbon monoxide has anti-inflammatory effects involving the mitogen-activated protein kinase pathway. *Nat. Med.* 6 422–428. 10.1038/74680 10742149

[B72] Pacifici F. Della-Morte D. Piermarini F. Arriga R. Scioli M. G. Capuani B. (2020). Prdx6 plays a main role in the crosstalk between aging and metabolic sarcopenia. *Antioxidants* 9:329. 10.3390/antiox9040329 32316601 PMC7222359

[B73] Penso-Dolfin L. Haerty W. Hindle A. Di Palma F. (2020). microRNA profiling in the Weddell seal suggests novel regulatory mechanisms contributing to diving adaptation. *BMC Genomics* 21:303. 10.1186/s12864-020-6675-0 32293246 PMC7158035

[B74] Pfluger P. T. Herranz D. Velasco-Miguel S. Serrano M. Tschöp M. H. (2008). Sirt1 protects against high-fat diet-induced metabolic damage. *Proc. Natl. Acad. Sci. U.S.A.* 105 9793–9798. 18599449 10.1073/pnas.0802917105 PMC2474520

[B75] Ponganis P. J. (2011). Diving mammals. *Compr. Physiol.* 1 447–465. 10.1002/cphy.c091003 23737181

[B76] Ponganis P. J. Kreutzer U. Sailasuta N. Knower T. Hurd R. Jue T. (2002). Detection of myoglobin desaturation in *Mirounga angustirostris* during apnea. *Am. J. Physiol. Regul. Integr. Comp. Physiol.* 282 R267–R272. 10.1152/ajpregu.00240.2001 11742847

[B77] Ponganis P. J. Kreutzer U. Stockard T. K. Lin P.-C. Sailasuta N. Tran T.-K. (2008). Blood flow and metabolic regulation in seal muscle during apnea. *J. Exp. Biol.* 211 3323–3332. 10.1242/jeb.018887 18840667

[B78] Pugh L. G. C. E. (1959). Carbon monoxide content of the blood and other observations on Weddell seals. *Nature* 183 74–76. 10.1038/183074a0 13622700

[B79] Pujade Busqueta L. Crocker D. E. Champagne C. D. McCormley M. C. Deyarmin J. S. Houser D. S. (2020). A blubber gene expression index for evaluating stress in marine mammals. *Conserv. Physiol.* 8:coaa082. 10.1093/conphys/coaa082 32904591 PMC7456562

[B80] R Core Team (2016). *RStudio: Integrated Development Environment for R.* Boston, MA: RStudio, Inc.

[B81] R Core Team (2019). *R: A Language and Environment for Statistical Computing.* Vienna: R Foundation for Statistical Computing.

[B82] Rangwala S. M. Wang X. Calvo J. A. Lindsley L. Zhang Y. Deyneko G. (2010). Estrogen-related receptor γ is a key regulator of muscle mitochondrial activity and oxidative capacity. *J. Biol. Chem.* 285 22619–22629. 10.1074/jbc.M110.125401 20418374 PMC2903389

[B83] Revelle W. (2019). *psych: Procedures for Psychological, Psychometric, and Personality Research. R Package Version 1.9.12.* Evanston, IL: Northwestern University.

[B84] Rhodes M. A. Carraway M. S. Piantadosi C. A. Reynolds C. M. Cherry A. D. Wester T. E. (2009). Carbon monoxide, skeletal muscle oxidative stress, and mitochondrial biogenesis in humans. *Am. J. Physiol. Heart Circ. Physiol.* 297 H392–H399. 10.1152/ajpheart.00164.2009 19465554 PMC2711725

[B85] Richmond J. P. Burns J. M. Rea L. D. (2006). Ontogeny of total body oxygen stores and aerobic dive potential in Steller sea lions (*Eumetopias jubatus*). *J. Comp. Physiol. B* 176 535–545. 10.1007/s00360-006-0076-9 16514541

[B86] Riddell J. R. Maier P. Sass S. N. Moser M. T. Foster B. A. Gollnick S. O. (2012). Peroxiredoxin 1 stimulates endothelial cell expression of VEGF via TLR4 dependent activation of HIF-1α. *PLoS One* 7:e50394. 10.1371/journal.pone.0050394 23185615 PMC3503895

[B87] Ryter S. W. (2020). Therapeutic potential of heme oxygenase-1 and carbon monoxide in acute organ injury, critical illness, and inflammatory disorders. *Antioxidants* 9:1153. 10.3390/antiox9111153 33228260 PMC7699570

[B88] Ryter S. W. Ma K. C. Choi A. M. K. (2018). Carbon monoxide in lung cell physiology and disease. *Am. J. Physiol. Cell Physiol.* 314 C211–C227. 10.1152/ajpcell.00022.2017 29118026 PMC5866434

[B89] Schmittgen T. D. Livak K. J. (2008). Analyzing real-time PCR data by the comparative CT method. *Nat. Protoc.* 3 1101–1108. 10.1038/nprot.2008.73 18546601

[B90] Seta F. Bellner L. Rezzani R. Regan R. F. Dunn M. W. Abraham N. G. (2006). Heme oxygenase-2 is a critical determinant for execution of an acute inflammatory and reparative response. *Am. J. Pathol.* 169 1612–1623. 10.2353/ajpath.2006.060555 17071585 PMC1780218

[B91] Sharick J. T. Vazquez-Medina J. P. Ortiz R. M. Crocker D. E. (2015). Oxidative stress is a potential cost of breeding in male and female northern elephant seals. *Funct. Ecol.* 29 367–376. 10.1111/1365-2435.12330 25983364 PMC4429057

[B92] Simonson T. S. Yang Y. Huff C. D. Yun H. Qin G. Witherspoon D. J. (2010). Genetic evidence for high-altitude adaptation in tibet. *Science* 329 72–75. 10.1126/science.1189406 20466884

[B93] Soriano-Arroquia A. Gostage J. Bardell D. McCloskey E. Bellantuono I. Clegg P. (2021). miR-24:Prdx6 interactions regulate oxidative stress and viability of myogenic progenitors during ageing. *bioRxiv* [Preprint]. 10.1101/2021.01.25.428069 PMC8520721 34560818

[B94] Stockard T. K. Levenson D. H. Berg L. Fransioli J. R. Baranov E. A. Ponganis P. J. (2007). Blood oxygen depletion during rest-associated apneas of northern elephant seals (*Mirounga angustirostris*). *J. Exp. Biol.* 210 2607–2617. 10.1242/jeb.008078 17644675

[B95] Suliman H. B. Carraway M. S. Tatro L. G. Piantadosi C. A. (2007). A new activating role for CO in cardiac mitochondrial biogenesis. *J. Cell Sci.* 120 299–308. 10.1242/jcs.03318 17179207

[B96] Sultanova R. F. Schibalski R. Yankelevich I. A. Stadler K. Ilatovskaya D. V. (2020). Sex differences in renal mitochondrial function: a hormone-gous opportunity for research. *Am. J. Physiol. Renal Physiol.* 319 F1117–F1124. 10.1152/ajprenal.00320.2020 33135479 PMC7792688

[B97] Sun J. Guo E. Yang J. Yang Y. Liu S. Hu J. (2017). Carbon monoxide ameliorates hepatic ischemia/reperfusion injury via sirtuin 1-mediated deacetylation of high-mobility group box 1 in rats. *Liver Transpl.* 23 510–526. 10.1002/lt.24733 28133883

[B98] Tan Q. Huang Q. Ma Y. L. Mao K. Yang G. Luo P. (2018). Potential roles of IL-1 subfamily members in glycolysis in disease. *Cytokine Growth Factor Rev.* 44 18–27. 10.1016/j.cytogfr.2018.11.001 30470512

[B99] Thorson P. H. Le Boeuf B. J. (1994). “Developmental aspects of diving in northern elephant seal pups,” in *Elephant Seals: Population Ecology, Behavior, and Physiology*, eds Le Boeuf B. J. Laws R. M. (Berkeley, CA: University of California Press), 271–289.

[B100] Tift M. S. Alves de Souza R. W. Weber J. Heinrich E. C. Villafuerte F. C. Malhotra A. (2020). Adaptive potential of the heme oxygenase/carbon monoxide pathway during hypoxia. *Front. Physiol.* 11:886. 10.3389/fphys.2020.00886 32792988 PMC7387684

[B101] Tift M. S. Ponganis P. J. (2019). Time domains of hypoxia adaptation—elephant seals stand out among divers. *Front. Physiol.* 10:677. 10.3389/fphys.2019.00677 31214049 PMC6558045

[B102] Tift M. S. Ponganis P. J. Crocker D. E. (2014). Elevated carboxyhemoglobin in a marine mammal, the northern elephant seal. *J. Exp. Biol.* 217(Pt 10) 1752–1757. 10.1242/jeb.100677 24829326 PMC4020943

[B103] Tift M. S. Ranalli E. C. Houser D. S. Ortiz R. M. Crocker D. E. (2013). Development enhances hypometabolism in northern elephant seal pups (*Mirounga angustirostris*). *Funct. Ecol.* 27 1155–1165. 10.1111/1365-2435.12111 24187422 PMC3811961

[B104] Tsuchiya M. Sekiai S. Hatakeyama H. Koide M. Chaweewannakorn C. Yaoita F. (2018). Neutrophils provide a favorable IL-1-mediated immunometabolic niche that primes GLUT4 translocation and performance in skeletal muscles. *Cell Rep.* 23 2354–2364. 10.1016/j.celrep.2018.04.067 29791847

[B105] Upadhyay K. K. Jadeja R. N. Vyas H. S. Pandya B. Joshi A. Vohra A. (2020). Carbon monoxide releasing molecule-A1 improves nonalcoholic steatohepatitis via Nrf2 activation mediated improvement in oxidative stress and mitochondrial function. *Redox Biol.* 28:101314. 10.1016/j.redox.2019.101314 31514051 PMC6737302

[B106] Ursini F. Maiorino M. (2020). Lipid peroxidation and ferroptosis: the role of GSH and GPx4. *Free Radic. Biol. Med.* 152 175–185. 10.1016/j.freeradbiomed.2020.02.027 32165281

[B107] Vázquez-Medina J. P. Zenteno-Savín T. Elsner R. (2006). Antioxidant enzymes in ringed seal tissues: potential protection against dive-associated ischemia/reperfusion. *Comp. Biochem. Physiol. C Toxicol. Pharmacol.* 142 198–204. 10.1016/j.cbpc.2005.09.004 16269268

[B108] Vázquez-Medina J. P. Zenteno-Savín T. Tift M. S. Forman H. J. Crocker D. E. Ortiz R. M. (2011a). Apnea stimulates the adaptive response to oxidative stress in elephant seal pups. *J. Exp. Biol.* 214(Pt 24) 4193–4200. 10.1242/jeb.063644 22116762 PMC3223118

[B109] Vázquez-Medina J. P. Olguín-Monroy N. O. Maldonado P. D. Santamaría A. Königsberg M. Elsner R. (2011b). Maturation increases superoxide radical production without increasing oxidative damage in the skeletal muscle of hooded seals (*Cystophora cristata*). *Can. J. Zool.* 89 206–212. 10.1139/z10-107 33356898

[B110] Vázquez-Medina J. P. Soñanez-Organis J. G. Burns J. M. Zenteno-Savín T. Ortiz R. M. (2011c). Antioxidant capacity develops with maturation in the deep-diving hooded seal. *J. Exp. Biol.* 214 2903–2910. 10.1242/jeb.057935 21832133 PMC3154117

[B111] Villena J. A. Hock M. B. Chang W. Y. Barcas J. E. Giguère V. Kralli A. (2007). Orphan nuclear receptor estrogen-related receptor α is essential for adaptive thermogenesis. *Proc. Natl. Acad. Sci. U.S.A.* 104 1418–1423. 17229846 10.1073/pnas.0607696104 PMC1783094

[B112] Vreman H. J. Wong R. J. Kadotani T. Stevenson D. K. (2005). Determination of carbon monoxide (CO) in rodent tissue: effect of heme administration and environmental CO exposure. *Anal. Biochem.* 341 280–289. 10.1016/j.ab.2005.03.019 15907874

[B113] Vreman H. J. Wong R. J. Stevenson D. K. (2001). “Sources, sinks, and measurement of carbon monoxide,” in *Carbon Monoxide and Cardiovascular Functions*, 1st Edn. ed. Wang R. (Boca Raton, FL: CRC Press), 274–310.

[B114] Vreman H. J. Wong R. J. Stevenson D. K. Smialek J. E. Fowler D. R. Li L. (2006). Concentration of carbon monoxide (CO) in postmortem human tissues: effect of environmental CO exposure. *J. Forensic Sci.* 51 1182–1190. 10.1111/j.1556-4029.2006.00212.x 17018107

[B115] Wegiel B. Otterbein L. E. (2012). Go green: the anti-inflammatory effects of biliverdin reductase. *Front. Pharmacol.* 3:47. 10.3389/fphar.2012.00047 22438844 PMC3306015

[B116] Weitzner E. L. Fanter C. E. Hindle A. G. (2020). Pinniped ontogeny as a window into the comparative physiology and genomics of hypoxia tolerance. *Integr. Comp. Biol.* 60 1414–1424. 10.1093/icb/icaa083 32559283

[B117] Wright T. J. Davis R. W. Holser R. R. Hückstädt L. A. Danesi C. P. Porter C. (2020). Changes in northern elephant seal skeletal muscle following thirty days of fasting and reduced activity. *Front. Physiol.* 11:564555. 10.3389/fphys.2020.564555 33123026 PMC7573231

[B118] Xie F. Xiao P. Chen D. Xu L. Zhang B. (2012). miRDeepFinder: a miRNA analysis tool for deep sequencing of plant small RNAs. *Plant Mol. Biol.* 80 75–84. 10.1007/s11103-012-9885-2 22290409

[B119] Yang D. Peng Y. Ouzhuluobu, Bianbazhuoma, Cui C. Bianba (2016). HMOX2 functions as a modifier gene for high-altitude adaptation in Tibetans. *Hum. Mutat.* 37 216–223. 10.1002/humu.22935 26781569

[B120] Yao H. Peterson A. L. Li J. Xu H. Dennery P. A. (2020). Heme oxygenase 1 and 2 differentially regulate glucose metabolism and adipose tissue mitochondrial respiration: implications for metabolic dysregulation. *Int. J. Mol. Sci.* 21:7123. 10.3390/ijms21197123 PMC7582259 32992485

