# Supplementary material for: Ontogeny of Carbon Monoxide-Related Gene Expression in a Deep-Diving Marine Mammal
Source: Front Physiol. 2021 Oct 21;12:762102. doi: 10.3389/fphys.2021.762102 (PMC8567018; doi:10.3389/fphys.2021.762102)
Supplement: Supplementary file 1 [file Data_Sheet_1.docx]

# Supplementary Material

## Supplementary Figures

**Supplementary Figure 1.**Correlogram showing significant (*p*< 0.05) Spearman correlation (*r*_S_, colored circles) between baseline expression of 13 genes in northern elephant seal skeletalmuscle. Circle color denotes direction of relationship (brown: positive, green: negative), while size of circles and color intensity denote magnitude of correlation.

**Supplementary Figure 2.**Correlogram showing significant (*p*< 0.05) Spearman correlation (*r*_S_, colored circles) between baseline expression of 10 genes in northern elephant seal whole blood. Circle color denotes direction of relationship (brown: positive, green: negative), while size of circles and color intensity denote magnitude of correlation.

## Supplementary Tables

**Supplementary Table1.**Age class, fasting state, sex, and year sampled for animals used in the study.

| **Animal ID** | **Age Class** | **Fasting State** | **Sex** | **Year Sampled** |
| --- | --- | --- | --- | --- |
| pup1 | pup | early | male | 2020 |
| pup2 | pup | early | female | 2020 |
| pup3 | pup | early | male | 2020 |
| pup4 | pup | early | male | 2020 |
| pup5 | pup | early | female | 2020 |
| pup6 | pup | early | female | 2020 |
| pup7 | pup | early | female | 2020 |
| pup8 | pup | late | male | 2020 |
| pup9 | pup | late | male | 2020 |
| pup10 | pup | late | male | 2020 |
| pup11 | pup | late | female | 2020 |
| pup12 | pup | late | male | 2020 |
| pup13 | pup | late | male | 2020 |
| pup14 | pup | late | male | 2020 |
| pup15 | pup | late | male | 2020 |
| juv2 | juvenile | early | female | 2017 |
| juv15 | juvenile | early | male | 2017 |
| juv16 | juvenile | early | male | 2017 |
| juv18 | juvenile | early | NA | 2017 |
| juv19 | juvenile | early | male | 2017 |
| juv20 | juvenile | early | female | 2017 |
| juv23 | juvenile | early | male | 2017 |
| juv28 | juvenile | early | female | 2017 |
| juv29 | juvenile | early | female | 2017 |
| fem_early_6 | adult | early | female | 2020 |
| fem_early_7 | adult | early | female | 2020 |
| fem_early_8 | adult | early | female | 2021 |
| fem_early_9 | adult | early | female | 2021 |
| fem_early_10 | adult | early | female | 2021 |
| fem_early_11 | adult | early | female | 2021 |
| fem_late_2 | adult | late | female | 2020 |
| fem_late_3 | adult | late | female | 2020 |
| fem_late_4 | adult | late | female | 2020 |
| fem_late_5 | adult | late | female | 2020 |
| fem_late_6 | adult | late | female | 2020 |
| fem_late_7 | adult | late | female | 2021 |
| fem_late_8 | adult | late | female | 2021 |
| male_early_1 | adult | early | male | 2020 |
| male_early_2 | adult | early | male | 2020 |
| male_early_3 | adult | early | male | 2020 |
| male_early_4 | adult | early | male | 2020 |
| male_early_5 | adult | early | male | 2020 |
| male_late_6 | adult | late | male | 2020 |
| male_late_7 | adult | late | male | 2020 |
| male_late_8 | adult | late | male | 2020 |
| male_late_10 | adult | late | male | 2020 |
| male_late_11 | adult | late | male | 2020 |
| male_late_12 | adult | late | male | 2020 |

**Supplementary Table 2.**Sequences and amplification efficiencies for qPCR primers used in the study. Primers were designed using sequences from NES muscle and blubber transcriptomes (Khudyakov et al., 2015; Deyarmin et al., 2019).

| **Gene** | **Transcript ID** | **Protein name** | **Primer sequence (5' to 3')** | **E%** |
| --- | --- | --- | --- | --- |
| *HMOX1* | TRINITY_DN582021_c0_g5 | Heme oxygenase 1 | F: CTGGTGATGGCCTCTCTTTACC  R: GCGTAGACCGGGTTGTCC | 97.8 |
| *HMOX2* | TRINITY_DN573552_c5_g3 | Heme oxygenase 2 | F: CACACTCGGCAACGATGTCA  R: CTCAGAGAGGTCAGCCATCCT | 99.5 |
| *BVR* | tr486081 | Biliverdin reductase | F: CTGGGTCTCTGGAGAATATGCC  R: GCCCAGGAGTTTCTGGACAA | 93.3 |
| *GPX3* | tr295337 | Glutathione peroxidase 3 | F: TAGCTGGCCACGTTGACAAA  R: GTGAGCGGCACCATCTATGA | 95.9 |
| *NRF2* | tr11361 | Nuclear factor erythroid 2-related factor | F: GCTGCGTTTCAGTCACTTGTT  R: GTTTGAGGTGACTGAGCCTGA | 96.9 |
| *PGC1A* | tr433035 | Peroxisome proliferator activated receptor gamma coactivator 1-alpha | F: GAGGAGGAGTTGTGGGTGGA  R: ACCCCAAGGGTTCCCCATTT | 87.3 |
| *IL1B* | tr50419 | Interleukin-1 beta | F: CCGTCGTCTGTGAGGTGTAT  R: ACAGCACCAGGGATTTTTGG | 101.6 |
| *ESRRA* | tr210045 | Estrogen-related receptor alpha | F: CACACCCAACACCAAGACCT  R: CAGGCTTCTCGTCACTGTCA | 84.0 |
| *ESRRG* | tr348270 | Estrogen-related receptor gamma | F: GTCCCCGACAGTGACATCAA  R: CCTGGAATATGCTTCGCCCA | 106.9 |
| *EF2* | tr388769 | Elongation factor 2 | F: TGGCCAAATTTGCTGCCAAG  R: CTTGCTGAATTTGCCGTTGG | 100.7 |
| *GAPDH* | tr223210 | Glyceraldehyde 3 phosphate dehydrogenase | F: CAGAACATCATCCCTGCCTC  R: TGCTTCACCACCTTCTTGA | 97.2 |
| *SIRT1* | tr23215 | NAD-dependent protein deacetylase sirtuin-1 | F: ACCACCAGATTCTTCAGCGAT  R: TTTGATTCGGACACACCAGGA | 90.6 |
| *PRDX1* | tr139285 | Peroxiredoxin 1 | F: GCTCTTCTGGACATCAGGCT  R: TGGTTCAGGCCTTCCAGTTT | 90.2 |
| *PRDX6* | tr94909 | Peroxiredoxin 6 | F: GCACCACAGAGCTTGGCAGA  R: AGGATGGCAAGGTCCCGATT | 95.6 |
| *GPX4* | tr448784 | Phospholipid hydroperoxide glutathione peroxidase | F: AAGTACCGGGGCTTCGTGTG  R: CCAGCGGCGAACTCTTTGAT | 93.8 |
| *IL10* | tr225261 | Interleukin 10 | F: AGAGGTGTCTACAAAGCCATGA  R: GTTTTGTTCCCCAGCCTGTTT | 100.8 |
| *CCL4* | TRINITY_DN588869_c10_g1 | C-C motif chemokine 4 | F: CACCGCCTGCTGCTTTTCTT  R: AGAGGCTGCTCGTCTCGAAG | 104.6 |
| *TLR4* | tr82854 | Toll-like receptor 4 | F: GGTGGCATTTGGCTCACTTC  R: TTGGAGGGAGAGGAGAGGTT | 92.8 |
| *TNFA* | TRINITY_DN591924_c5_g5 | Tumor necrosis factor | F: TGGAATCATTGCCCTGTGAGG  R: CTAAGCCAGAAGGGGATGAGG | 95.0 |

**Supplementary Table3.**PCA diagnostics (Field et al., 2012) for skeletal muscle and whole blood gene expression datasets.

|  | Muscle PCA | Blood PCA |
| --- | --- | --- |
| Number of genes | 13 | 10 |
| Determinant | 0.0001 | 0.0002 |
| Barlett’s test | 𝛘^2^ (78) = 364.03  p < 0.0001 | 𝛘^2^ (45) = 237.91  p < 0.0001 |
| KMO test | overall MSA = 0.73 | overall MSA = 0.73 |
| Mean communality | 0.77 | 0.79 |
